# Convergence of Nanotechnology and Bacteriotherapy for Biomedical Applications

**DOI:** 10.1002/advs.202309295

**Published:** 2024-02-15

**Authors:** Jun Liu, Sichen Yuan, Alexa Bremmer, Quanyin Hu

**Affiliations:** ^1^ Pharmaceutical Sciences Division, School of Pharmacy University of Wisconsin, Madison (UW‐Madison) Madison WI 53705 USA; ^2^ Wisconsin Center for NanoBioSystems University of Wisconsin, Madison (UW‐Madison) Madison WI 53705 USA; ^3^ Carbone Cancer Center, School of Medicine and Public Health University of Wisconsin, Madison (UW‐Madison) Madison WI 53705 USA

**Keywords:** bacteriotherapy, diagnosis, nanotechnologies, therapy

## Abstract

Bacteria have distinctive properties that make them ideal for biomedical applications. They can self‐propel, sense their surroundings, and be externally detected. Using bacteria as medical therapeutic agents or delivery platforms opens new possibilities for advanced diagnosis and therapies. Nano‐drug delivery platforms have numerous advantages over traditional ones, such as high loading capacity, controlled drug release, and adaptable functionalities. Combining bacteria and nanotechnologies to create therapeutic agents or delivery platforms has gained increasing attention in recent years and shows promise for improved diagnosis and treatment of diseases. In this review, design principles of integrating nanoparticles with bacteria, bacteria‐derived nano‐sized vesicles, and their applications and future in advanced diagnosis and therapeutics are summarized.

## Graphical abstract

Nanoparticles and bacteria integration holds promise for diverse biomedical applications. This review summarizes strategies and design principles to construct effective delivery/therapeutic platforms combining nanoparticles and bacteria, with a focus on emerging disease diagnosis and treatment applications. Furthermore, the microbiota modulation effect of nanoparticles, the challenges, and perspectives are discussed with a highlight on the future development of novel delivery/therapeutic systems.

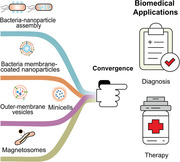

## 1 Introduction

Bacteria are the most important species of microorganisms in gut microbiota; thus, they have a profound impact on human health and disease.^[^ ^1^, ^2^ ^]^ Bacteria possess a range of properties that make them suitable for use in the diagnosis and treatment of various diseases. Due to their abilities to sense, respond, and facilitate balance of gut microbiota, bacteria have been directly used as therapeutics. For instance, the fecal microbiota transplantation for drug‐refractory *Clostridium difficile* infection.^[^ ^3^, ^4^ ^]^ Recently, the potential of bacteriotherapy has been explored more thoroughly. Properties, including biocompatibility, tropism, and motility, position bacterial as an ideal candidate for the engineering of an intelligent drug delivery platform. For instance, tropism toward chemical gradients, light, or even external magnetic fields efficiently orient bacteria to sense and respond to different stimulus in the surroundings. Other beneficial characteristics are apparent with independent movement and colonization driven by rotating flagella and pili.^[^ ^5^, ^6^, ^7^, ^8^ ^]^ Thesse inherent properties may be utilized for recognizing, targeting, and accumulating purposes for drug delivery.^[^ ^9^ ^]^ Furthermore, the applications of bacteria‐based technology are not limited to novel therapies but are also actively involved in imaging, vaccine development, and diagnosis.

Despite the numerous advantages that make bacteria an appealing choice for treating a range of medical conditions and diseases, there are still significant challenges. One such challenge is the oral administration of probiotics, a commonly used route for bacteriotherapy, where the viability of bacteria is severely compromised due to the acidic stomach environment and the presence of bile salts. Furthermore, the short retention time of bacteria in the intestines leads to inadequate accumulation and dosage of therapeutics, resulting in suboptimal treatment efficacy. In addition, while bacteria exhibit tropism toward various stimuli, bacteria‐based therapeutics often lack robust targeting capabilities and specificity, necessitating further refinement.

Fortunately, the convergence of nanotechnologies and bacteriotherapy has significantly improved the survival and resistance of bacteria‐based therapeutics in harsh environments, enhanced their spatiotemporal specificity, and equipped bacteria with additional properties for interaction and adaptation to their surroundings. The combination of nanotechnology and bacteriotherapy can be accomplished through various methods. One approach involves using the bacterial membrane to encapsulate nanoparticles while another is generating bacteria‐nanoparticle hybrids through multiple modification strategies. Among those strategies, chemical engineering, genetic engineering, and bioconjugation through non‐covalent/covalent binding and electrostatic interactions are commonly employed for cell engineering. Additionally, the innovative concept of biomineralization has been explored as a distinctive approach to engineer bacteria and create bacteria‐derived nanoparticles. The abundance of options for bacteria modification significantly contributes to the versatility of bacteriotherapy, including the creation of various types of bacterial minicells and membrane vesicles. Therefore, a wealth of tools and design strategies have been applied in areas such as diagnosis, imaging, and therapies including immunotherapy.

**Figure 1:**
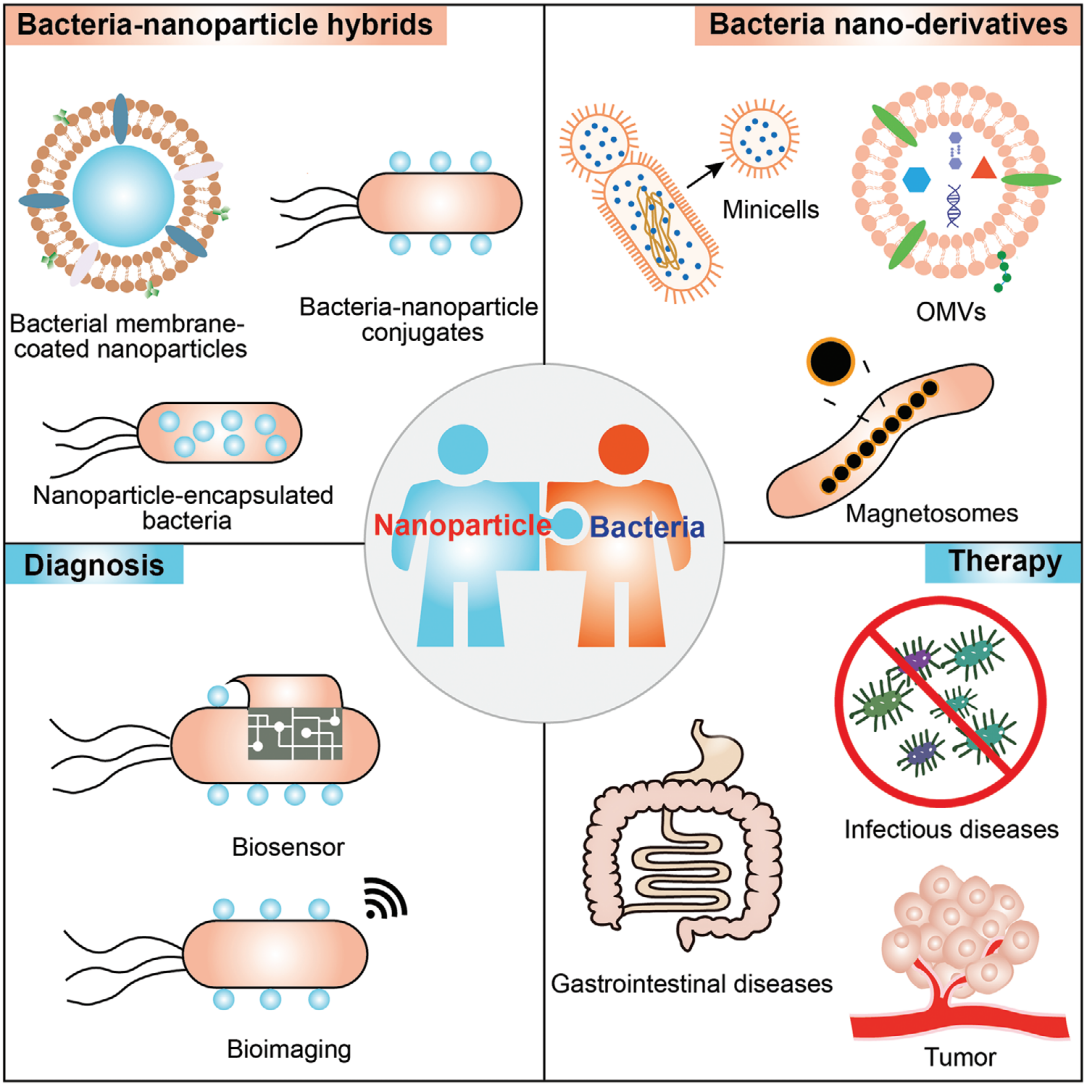
Schematic illustration of the fundamental and design principle of convergence nanotechnology with bacteria for diseases diagnosis and therapy.

In this review, we summarize the current strategies of integrating nanoparticles with bacteria and bacteria‐derived nanovesicles to clarify the design principles for the delivery system. The emerging applications in diagnosis and therapeutics and the impact of nanoparticles for microbiota modulation, future challenges, and prospects are outlined and discussed (**Figure** **1**).

## 2 Design Principles for Convergence of Nanotechnology and Bacteriotherapy

### 2.1 Bacteria Membrane‐Encapsulated Nanoparticle

**Figure 2:**
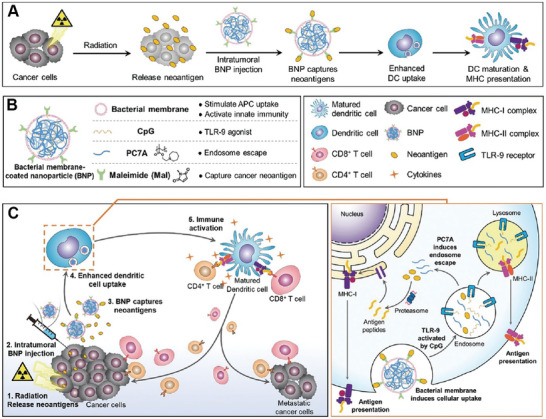
Bacterial membrane‐encapsulated nanoparticles for antitumor immune responsive activation. A) Schematic illustration of the interaction of bacterial membrane‐encapsulated nanoparticles with tumor microenvironments to enhance antigen‐presenting cells’ uptake and activation. B) The formation of bacterial membrane‐encapsulated nanoparticles and the function of each component. C) Schematic illustration of antitumor immune activation by combined bacterial membrane‐encapsulated nanoparticles with radiation treatment. Reproduced with permission.^[^ ^11^ ^]^ Copyright 2019, Wiley‐VCH.

The membrane of bacteria is abundant in lipopolysaccharides and pathogen‐associated molecules, making them immunogenic and capable of provoking and stimulating immune responses.^[^ ^10^ ^]^ Bacterial membrane also facilitates the uptake by various immune cells, which has drawn considerable attention for membrane encapsulation to aid immune response in cancer therapy and vaccine. The lack of specific tumor antigens and low expression of major histocompatibility complex molecules impede the recognition of tumor cells by T cells. Most notably recognition for cold tumors that are characterized by an immunosuppressive tumor microenvironment and present extremely poor response to immunotherapy. Combined with radiotherapy, bacterial membrane encapsulation could facilitate the recognition of released tumor neoantigens by radiotherapy, while promoting maturation of dendritic cells (DCs) and antigen uptake for cross‐presentation of antigens in adaptive immunity activation (**Figure** **2**A). Patel et al. used imide groups‐modified bacterial membrane to encapsulate nanoparticles consisting of anionic cytosine, guanine nucleotides (CpG) and poly(ethylene glycol)‐b‐poly(2‐(hexamethyleneimino)ethyl methacrylate) (PC7A) following radiotherapy (Figure 2B).^[^ ^11^ ^]^ The modification of maleimide group on bacterial membrane surface facilitate the capture of release neoantigens. Taking advantage of the bacterial membrane, the dendritic cells within tumor elicit higher uptake efficiency of nanoparticles, allowing the subsequent toll‐like receptor‐9 (TLR9) activation and antigen cross‐presentation. This bacterial membrane encapsulated nanoparticles combined with radiation therapy significantly regressed tumors, increased survival rate, and achieved a long‐term anti‐tumor immune memory (Figure 2C). Despite of DCs, the propensity of neutrophils to engulf bacteria also makes the bacterial membrane ideal for creating biohybrid neutrophil micromotors for targeted drug delivery.^[^ ^12^ ^]^ After being encapsulated in the bacterial membrane, mesoporous silica nanoparticles were able to hitchhike neutrophils through specific uptake, thereby generating chemotaxis‐guided and nanoparticle‐loaded live micromotor. The bacterial membrane helps to protect nanoparticles inside neutrophils from undesired release while preserving neutrophils’ bioactivity and chemotaxis capability.

However, the endotoxins that the bacterial wall contains after bacterial membrane coating can cause serious adverse effects such as cytokine storm and sepsis. To solve this problem, a bacterial cytoplasmic membrane isolated from bacteria after removing lipopolysaccharides (LPS) and other endotoxins is used to reduce the toxic effects while still acting as an adjuvant to stimulate immune responses. Chen et al. created a hybrid membrane by fusing a tumor cell membrane and a bacterial cytoplasmic membrane. This hybrid membrane was used to encapsulate poly(lactic‐co‐glycolic acid) (PLGA) nanoparticles, which can simultaneously deliver antigens and adjuvants inherited from both sources of cell membranes, thus enhancing innate immune response and maximizing anti‐tumor effects while avoiding side effects.^[^ ^13^ ^]^

### 2.2 Bacteria‐Nanoparticle Hybrids

Bacteria‐nanoparticle hybrids represent an exciting new technology, which combines the biological properties of bacteria with the material functions of nanoparticles, broadening the range of drugs in the bacteriotherapy. Various methods have been developed to generate and design bacteria‐nanoparticle hybrids, such as bioconjugation, electrostatic interactions, chemical conjugation, and biomineralization. Here, we summarized the typical technologies utilized to combine bacteria and nanotechnology and the resulting biological functions.

#### 2.2.1 Non‐Covalent Binding

##### Bioconjugation

**Figure 3:**
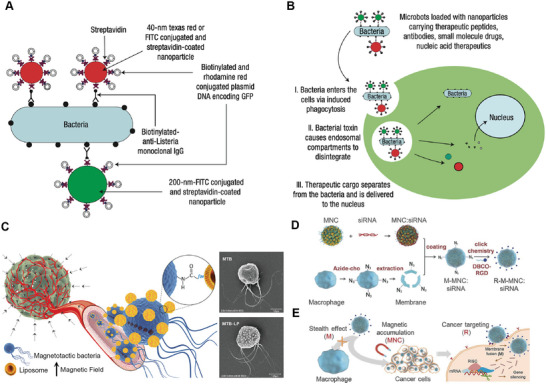
Representative strategies for convergence bacteria with nanoparticles. A) Conjugation of bacteria with functional polystyrene nanoparticles through streptavidin‐biotin binding. B) Illustration of the formed bacteria‐nanoparticle hybrid be internalized into cells and releases payloads. Reproduced with permission.^[^ ^15^, ^43^, ^44^ ^]^ Copyright 2007, Springer Nature. C) Conjugating carbonylated liposomes on the surface of magnetotactic bacteria via covalent binding. The hybrid exhibits tumor‐targeting under an external magnetic field. Reproduced with permission.^[^ ^27^ ^]^ Copyright 2014, American Chemical Society. D) Combination of multiple binding strategies for functionalizing biomineralized magnetic nanoparticles from bacteria for efficient siRNA delivery, including azide‐membrane engineering, electrostatic interaction, and click reaction. E) The functionalized magnetosome shows tumor targeting effect and effective siRNA delivery for antitumor therapy. Reproduced with permission.^[^ ^41^ ^]^ Copyright 2018, Wiley‐VCH.

Streptavidin and biotin are one of the most used non‐covalent conjugation couples in biotechnological applications due to their highly selective and robust interaction. Streptavidin is a 56‐kDa homotetramer produced by the bacterium *streptomyces avidinii* that binds up to four biotin molecules. A strong, non‐covalent bond is formed when the positively charged lysine group of streptavidin interacts with the negatively charged carboxyl group of biotins, developing a stable and highly efficient complex. The interaction is so strong that it can withstand extreme temperatures (*T*_m_ of 112 °C), pH, denaturing agents, and enzymes, making it an ideal choice for a wide range of applications.^[^ ^14^ ^]^ The strategy of utilizing the interaction between streptavidin and biotin is highly effective for attaching nanoparticles of various sizes and different entities, including peptides, antibodies, small molecule drugs, and nucleic acid therapeutics, to bacteria. These hybrid structures are known as bacteria‐nanoparticle hybrids. For example, nanoparticles coated with streptavidin and bacteria with biotinylated monoclonal antibody C11E9 modified through *N*‐acetylmuramidase were conjugated via streptavidin‐biotin binding (**Figure** **3**A).^[^ ^15^ ^]^ After the internalization of bacteria‐nanoparticle hybrid through a process known as bactofection, cargo release can be further induced by endosomal compartments and bacterial toxins (Figure 3B).^[^ ^16^ ^]^ Monovalent streptavidin with a single femtomolar biotin binding site, which has been modified to maintain the affinity and thermostability of wild‐type streptavidin, has also been developed for other applications such as single‐particle tracking.^[^ ^17^, ^18^ ^]^ Furthermore, liposome, a nanoparticle used for drug delivery, can restrict the biotin site by altering the amount and position of raft domains, allowing for a tunable binding configuration of streptavidin‐biotin complex.^[^ ^19^ ^]^ This improves the motility of hybrid, thereby enhancing drug delivery.

Besides attaching nanoparticles to bacteria through the streptavidin‐biotin interaction, genetic modification of bacteria expressing surface‐displayed peptides represents another approach for bioconjugation. Genetic engineering bacteria enables the manipulation of peptides on the cell surface to create desirable effects, such as increasing the binding affinity and introducing new binding sites that preferentially attach specific nanoparticles. For example, the sequences for expression of histidine and methionine on cell surfaces have been shown to have an affinity to metal nanoparticles and quantum dots.^[^ ^6^ ^]^ Furthermore, the rational design of gene circuits allows for the controlled binding of nanomaterials while preserving cell viability and proliferation. Genetic modification for bioconjugation exhibits high loading capacity and can overcome the repellent forces between negatively charged nanoparticles and negative cell surfaces, making it a promising platform for a wide range of applications.^[^ ^7^ ^]^

##### Electrostatic Interaction

Due to the negative charge of most bacterial surfaces, electrostatic interaction is a commonly used method to deposit cationic nanoparticles to bacteria. Polyethyleneimine (PEI) is a widely used cationic polymer with a strong buffering capacity, allowing it to escape from endo/lysosome due to the “proton sponge” effect. PEI spontaneously polymerizes with DNA to form nanoparticles, which can then be electrostatically deposited on bacterial surfaces. This strategy is straightforward and does not require any surface premodification of bacteria or nanoparticles. The living hybrid, loaded with antigen‐expressing plasmid, is an effective DNA vaccine delivery platform, where bacteria facilitate the hybrid uptake by cells; simultaneously, PEI/DNA nanoparticle coating improves the gastric acid tolerance of bacteria after oral delivery and allows for phagosome escape, enabling antigen expression to modulate immune responses.^[^ ^20^ ^]^

Maghemite nanoparticles with a positive charge can be easily loaded onto bacterial surfaces through agitation, creating magnetotactic bacteria without compromising their viability and proliferation.^[^ ^21^ ^]^ During bacterial division, magnetic nanoparticles are equally distributed among the offspring bacteria. These living magnets can be used in various biomedical applications, including magnetic resonance imaging and hyperthermia.^[^ ^22^ ^]^

Anionic materials are also used to form nanoparticles, so it is important to consider ways to interact with anionic nanoparticles and bacteria. Alternatively, a positively charged layer can be used to deposit anionic nanoparticles on bacteria, avoiding direct contact with the bacterial membrane. For instance, to attach citrate‐reduced Au/Ag to the bacterial membrane, poly (allylamine hydrochloride) (PAH), a positive‐charged polymer, is encapsulated on the bacterial surface through electrostatic interaction. Additionally, a layer of PAH is applied to the outer surface of Au and Ag nanoparticles to enhance stability.^[^ ^23^ ^]^ This layer‐by‐layer strategy has been proven to be a highly effective method for coating various surfaces, as it is relatively simple to prepare via electrostatic interactions. Similarly, multiple bilayers can be generated to encapsulate probiotics using cationic polysaccharide chitosan and anionic polysaccharide alginate which both are biocompatible and have mucoadhesive properties. Encapsulated probiotics have shown increased survivability against gastric acid and bile salt assaults, as well as a longer retention time in intestinal tissues.^[^ ^24^ ^]^ The electrostatic interaction could be utilized with almost any charged nanoparticles, polyelectrolyte, proteins, or polysaccharides. In a recent study, norepinephrine is used to encapsulate probiotics for robust mucosa adhesion and provides anchoring for the polymer of hyaluronic acid‐poly (propylene sulfide) (HA‐PPS) to function as reactive oxygen species scavengers.^[^ ^25^ ^]^ Moreover, electrostatic interaction allows for nanoparticles to be customized and functionalized for different delivery applications, thereby expanding potential applications.

In addition to the mentioned materials, the alteration of the zeta potential of bacteria precedes the potential utilization of negatively charged nanoparticles, presenting an alternative approach for electrostatic absorption to bacteria. The surrounding environment could significantly influence the balance of positively and negatively charged groups on the bacterial surface. For example, following a 15‐minute incubation with a 12.5 m CaCl_2_ solution, the zeta potential of probiotic *Escherichia coli 1917* (EcN) underwent a substantial shift from −18.4 to +5.16 mV. This conversion facilitates the subsequent electrostatic adherence of hyaluronidase‐hybridized nanoparticles.^[^ ^26^ ^]^

#### 2.2.2 Chemical Conjugation

Chemical conjugation is generally considered to be more stable than non‐covalent binding. Chemical groups on the surface of bacteria make it possible for bacteria and nanoparticle conjugation. Considering the presence of amine groups on the surface of bacteria, it is possible to use these amine groups as the docking site for nanoparticle bioconjugation. As a strong nucleophile, amine readily reacts with electrophiles, such as carboxyl groups to form amides, unsaturated ketone groups to undergo Michael addition, and carbonyl groups to create Mannich bases. Based on these chemical properties, Taherkhani et al. used carbodiimide cross‐linking chemistry to attach carboxylated nanoliposomes to magnetotactic bacteria, creating self‐propelled therapeutic agents (Figure 3C).^[^ ^26^, ^27^ ^]^ Under the control of an external magnetic field, this bacteria‐nanoliposome hybrid with a high payload capability can selectively and efficiently transport drug‐loaded liposomes to tumor sites, resulting in enhanced anti‐tumor efficacy and minimal toxicity.

Click reaction is another method for covalently bonding nanoparticles to live bacteria, which is highly specific, selective, and orthogonal. For instance, copper‐free azide‐alkyne cycloaddition enables *E. coli* bacteria decorated with azide groups to covalently bind to mesoporous silica nanoparticles decorated with alkynes. These bacteria can transport nanoparticles deep into tumor tissues, taking advantage of the aerotaxis property of anaerobic bacteria, and thus facilitate improved release of antitumor drugs to tumor cells.^[^ ^29^ ^]^ Using chemical technologies, the thiol group could also be converted from amine group by chemical reactions for further conjugation.^[^ ^30^ ^]^ In general, carboxyl groups, amine groups, and azide groups are commonly employed as binding sites for covalent conjugation, offering flexibility based on nanoparticle properties. However, the efficiency of reactions involving these chemical groups may vary across different bacterial strains. Further investigation is warranted to characterize the density of reactive groups on bacteria surface, and the development of new methods with enhanced conjugation efficiency should be pursued.

#### 2.2.3 Biomineralization

Biomineralization is a process of utilizing mineral elements by living organisms, which is a promising approach for material engineering. Bacteria, due to their genetically manipulable nature, are used as a model system to create organic–inorganic hybrids with novel features and functions that are not achievable by abiotic materials. Different from attaching and adding nanoparticles on the bacterial surface, this method can help to directly form nanoparticles by bacteria themselves. There are mainly two types of biomineralization processes: biologically induced and biologically controlled. The biologically induced process is not directly regulated by genes, and mineralization usually occurs on the surface of bacteria. In contrast, the processes of biologically controlled mineralization, including nucleation, growth, and location, can be genetically regulated and take place intracellularly and extracellularly. Notably, the supersaturated mineral solution is required for nuclei formation. There are several examples of biologically induced biomineralization. When *S. algae* K3259 was incubated under the conditions of HAuCl_4_ and sodium lactate, gold nanoparticles were mineralized on the bacterial surface. These gold nanoparticles can act as photoexcited electron donors, transferring optical energy to electrons to expedite bacterial metabolic activities.^[^ ^31^ ^]^ When Cd^2+^ and cysteine were added as the sulfur source, cadmium sulfide nanoparticles were biologically precipitated on the surface of *Moorella thermoacetica* and functioned as the light harvester to support cellular metabolism.^[^ ^32^ ^]^ Furthermore, metal‐organic frameworks (MOFs), which are metal ions/clusters crosslinked with organic linkers with excellent biocompatibility, have also been generated by biomimetic mineralization without having a negative impact on bacterial viability and tumor‐targeting ability.^[^ ^33^ ^]^

As for biologically controlled biomineralization, magnetic nanoparticles called magnetosomes produced by magnetotactic bacteria are a typical paradigm. The magnetosomes are organized in chains that passively align themselves along the magnetic field lines of the earth; therefore, the bacteria can be manipulated by an external magnetic field enabling their location to be determined by magnetic resonance imaging.^[^ ^33^, ^34^ ^]^ Furthermore, the properties of magnetosomes, such as size, surface area, chain length, and number of superparamagnetic particles, can be modified through genetic engineering.^[^ ^35^, ^36^ ^]^ Plasmids containing magnetosome gene operons can be integrated into chromosomes by endogenous transposases in magnetotactic bacteria for controlling magnetosome production.^[^ ^38^ ^]^ For example, increasing the expression of MamC, a *M. magneticum* gene that affects the size of iron oxide nanoparticles, regulates the size and shape of magnetosomes. Controlling the overexpression of Mms6 promotes the production of smooth and spherical particles, while MamK overexpression leads to more long‐chain magnetosomes.^[^ ^39^ ^]^ Deleting a *fur*‐like gene required for ferric uptake reduces biomineralization and increases the level of free intracellular iron.^[^ ^40^ ^]^ Similar to strategies used for bacterial membrane modification, magnetosome membranes can also be biofunctionalized for various biomedical applications. Zhang et al. created magnetic nanoclusters decorated with PEI as a surfactant, enabling siRNA binding via electrostatic interactions (Figure 3D).^[^ ^41^ ^]^ The formed complex was then camouflaged with macrophage membranes, prolonging its circulation in the bloodstream for siRNA delivery. The functionalized magnetosomes could target tumor tissues under the guidance of tumor targeting peptide Arg‐Gly‐Asp and external magnetic control, thus achieving higher selectivity and targeting for enhanced antitumor therapy (Figure 3E). Using the EDC/NHS method, the magnetosome membrane can be modified with molecules such as a bacteria‐derived chemotactic peptide, promoting site‐specific immune cell uptake and initiating chemotactic recruitment of circulating immune cells.^[^ ^42^ ^]^ T‐cell stimuli or cancer cell membranes might be decorated on magnetosomes, and serve as artificial antigen‐presenting cells to promote antigen‐specific T‐cell expansion. Additionally, due to their superparamagnetism and magnetization characteristics, magnetosomes can be directed to and retained in lymph nodes by magnetic resonance imaging, thus facilitating antigen uptake by dendritic cells.

### 2.3 Bacterial Derived Nanovesicles

#### 2.3.1 Bacterial Minicells

Minicells are small bacterial cells (≈400 nm diameter) produced during abnormal bacterial division, which are genetically controlled by the mutant *minCDE* gene. Bacterial minicells contain almost all cellular components, except for chromosomal DNA, thereby preventing them from proliferating. Bacterial minicells are powerful for drug delivery because of their non‐active property and the ability to package various drugs by unidirectional diffusion, regardless of their physicochemical properties.^[^ ^44^, ^45^, ^46^ ^]^ Their surface is also easily modified with antibodies or ligands for targeting. For example, the surface of minicells can be coupled with bispecific antibodies, in which one arm binds to lipopolysaccharide on the surface of minicells, and the other recognizes a specific receptor on the target cells, such as epidermal growth factor receptor (EGFR). After receptor‐mediated endocytosis into targeted cells, the minicells are degraded, following the release of payloads. Minicells equipped with targeting ability can be used to solve the problem of severe toxic side effects induced by systemic administration of therapeutic drugs. MacDiarmid et al. modified minicell surface with EGFR or human epidermal growth factor receptor‐2 (HER2) antibody, giving the minicells the ability to target EGFR or HER2 receptor specifically overexpressed on tumor cell membranes. After packaging therapeutic drugs of doxorubicin (DOX) and paclitaxel (PTX), these modified minicells carried drugs directly into targeted tumor cells. Minicells surface expressing a pH insertion peptide via genetic engineering can also target the acidic microenvironment of tumors.^[^ ^47^ ^]^ The anti‐tumor effects of this form of targeted delivery of therapeutic agents were more potent than free drugs even with thousands‐fold higher amounts and present negligible adverse side effects.^[^ ^47^, ^48^ ^]^ Currently, ^EGFR^minicells_PTX_ entered in a phase I clinical trial were proven safe in patients.^[^ ^50^ ^] EGFR^minicells_Dox_ was also studied in recurrent glioblastoma on humans with excellent safety profile.^[^ ^51^ ^]^

Except for conventional therapeutical drugs, bacterial minicells processes have advantages for RNAi delivery, such as efficient loading of siRNA (≈12 000 copies) and shRNA (≈100 copies), stability against the external environment, and robust targeting capability.^[^ ^52^ ^]^ MacDiarmid et al. reported an approach for delivering si/shRNA to treat cancer. The minicells were modified by bispecific antibodies to target tumor‐cell‐surface receptors, and the loaded si/shRNA could effectively inhibit cell cycle‐associated protein expression to induce cell cycle arrest and apoptosis in tumor cells. In addition to bispecific antibodies modification, ligands of various receptors on tumor cells are also common choices for minicells modification to achieve targeted delivery.^[^ ^53^ ^]^

One prerequisite for utilizing bacterial minicells is to purify them from parent bacteria and empty their cytosol contents. Purification of minicells is usually done through differential centrifugation, density gradient centrifugation, or filtration. However, these methods are challenging to eliminate parent bacteria and yield low results.^[^ ^53^, ^54^ ^]^ Optimization of purification methods has been investigated. For instance, the combination of antibiotics treatment with filtration or centrifugation can kill bacteria but not harm non‐dividing minicells, thus achieving higher yield.^[^ ^55^, ^56^ ^]^ Park et al. utilized the lytic proteins leading to the autolysis of parent bacteria, to have a high purity of minicells after density gradient centrifugation.^[^ ^58^ ^]^ Most novel purification methods rely on the different responses of minicells and parent bacteria toward treatment.

#### 2.3.2 Bacterial Membrane Vesicles

Bacterial membrane vesicles (MVs) are nanovesicles released from both Gram‐negative bacteria and Gram‐positive bacteria, with sizes ranging from ≈20 to 400 nm.^[^ ^59^ ^]^ They are protrusions pinched off from the outer membrane of Gram‐negative bacteria and are often referred to as outer‐membrane vesicles (OMVs).^[^ ^59^, ^60^ ^]^ MVs were first discovered in the 1960s and believed to be secreted exclusively by Gram‐negative bacteria because the thick peptidoglycan layer of Gram‐positive bacteria was postulated to prevent MVs production. However, in 2009, MVs from the surface of a Gram‐positive bacteria, *Staphylococcus aureus*, were confirmed using electron microscopy.^[^ ^62^ ^]^ Thus, for clarity, we refer to MVs from Gram‐positive bacteria as inner‐membrane vesicles (IMVs) in this review. OMVs and IMVs have distinct structures and compositions. OMVs are composed of a single layer of phospholipid bilayer membrane enriched with periplasmic luminal components, like lipopolysaccharides, peptidoglycan, enzymes, outer‐membrane proteins, and nucleic acids.^[^ ^63^ ^]^ They can be secreted during normal bacteria growth without impact on membrane stability.^[^ ^64^ ^]^ In contrast, IMVs are composed of two phospholipid bilayers, lacking lipopolysaccharides, but containing cytoplasmic components. This is due to the single inner membrane of Gram‐positive bacteria, which is covered by a thick peptidoglycan layer without lipopolysaccharides.^[^ ^65^ ^]^ The formation of IMVs may be attributed to the expression of endolysin, which damages peptidoglycan and leads to the loss of membrane integrity and eventually cell death.^[^ ^66^ ^]^ Moreover, the numbers of MVs and their compositions vary between different bacteria or under different conditions, due to genetic and environmental modulation. Generally, MVs released from commensal bacteria can interact with mammalian cells to support and benefit the host's health. For instance, MVs containing polysaccharides released from *B. fragilis* can modulate immune responses by up‐regulating T cells and promoting anti‐inflammatory cytokines production to prevent diseases.^[^ ^67^ ^]^ MVs containing eukaryotic enzyme inositol phosphatase, secreted by *B. thetaiotaomicron*, may mediate intracellular calcium ion signaling to regulate human gastrointestinal physiology.^[^ ^68^ ^]^ On the contrary, MVs containing virulence factors and cytotoxins from pathogenic bacteria are believed to have evolved for invading and infecting hosts.

**Figure 4:**
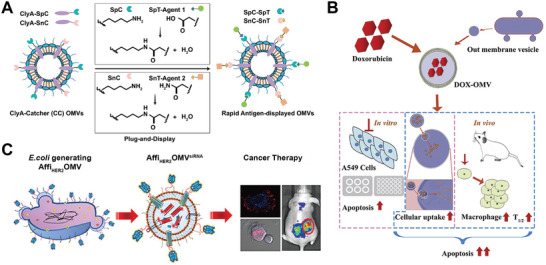
A) OMVs‐based antigen display platform for tumor vaccination via plug‐and‐display technology. Reproduced with permission.^[^ ^72^ ^]^ Copyright 2021, Springer Nature. B) OMVs serve as a chemotherapy drug carrier and synergize with their immunogenicity for tumor chemoimmunotherapy. Reproduced with permission.^[^ ^77^ ^]^ Copyright 2020, Elsevier B.V. C) Schematic illustration of siRNA‐loaded and engineered OMVs expressing HER2‐specific affibody for cancer therapy. Reproduced with permission.^[^ ^78^ ^]^ Copyright 2014, American Chemical Society.

Bacteria membrane vesicles are associated with numerous principal biological processes and are pivotal messengers for cell‐to‐cell communication. These vesicles can transport their contents, including virulence factors, cytotoxins, proteins, and genes, to neighboring cells without being damaged by host proteases, DNase, or antibodies. Due to their unique natural compositions, MVs possess inherent immunostimulatory properties, lymph node targeting, and can serve as immunotherapeutic agents for many diseases. By loading cargos that can co‐stimulate CD4^+^ and CD8^+^ T cells, MVs escape from the endosome to enter the cytoplasm, where they enable cross‐presentation, stimulate innate immunity, and promote adaptive immune responses.^[^ ^68^, ^69^ ^]^ Surface decoration for MVs with exogenous antigens is also accessible.^[^ ^71^ ^]^ Cheng et al. chose the transmembrane protein cytolysin A on MVs as an anchor site, and fused it with various tumor antigens by employing the protein plug‐and‐display system, including a SpyTag/SpyCatcher pair and a SnoopTag/SnoopCatcher pair (**Figure** **4**A).^[^ ^72^ ^]^ The Hemoglobin protease (Hbp) autotransporter platform was also developed to display heterologous polypeptides on the surface of MVs for multiple antigens fusion.^[^ ^72^, ^73^ ^]^ The simultaneous expression of multiple tumor antigens on MVs facilitates a synergistic anti‐tumor immune response, which is valuable for treating complex and heterogeneous tumors. Another strategy for antigen delivery by MVs is to express antigens in the bacterial periplasm and trap them in MVs lumen during vesicle formation.^[^ ^75^ ^]^ Different antigens, including *Group A Streptococcus* (GAS) Slo, SpyCEP, and Spy0269, together with OmpA, leader sequence for OMVs secretion, were fused into *E. coli*, and created a complex that exhibited immunization against GAS lethal challenge. Muralinath et al. also engineered a *Salmonella enterica* serovar Typhimurium strain that secretes pneumococcal protein PspA in the periplasm. MVs released from this strain contained PspA in the lumen, which could induce an immune response against infection.^[^ ^76^ ^]^

The abilities of MVs to transport drugs and to induce immune responses can be combined for an effective drug delivery system. For instance, OMVs released from *Klebsiella pneumonia* could encapsulate doxorubicin and deliver the drug into lung tumor cells. OMVs immunogenicity enable them to recruit macrophages in the tumor microenvironment. The delivered doxorubicin and the recruitment of macrophages by OMVs generated a synergistic effect, leading to improved tumor chemoimmunotherapy (Figure 4B).^[^ ^77^ ^]^ The targeting capability after antibody modification can also be combined with other therapeutical agents. Engineered OMVs secreted from ClyA‐affibody‐overexpressing *E. coli* expressed an anti‐HER2 affibody on the surface providing a carrier for siRNA that can lead to tumor regression by kinesin spindle protein (KSP) silencing (Figure 4C).^[^ ^78^ ^]^

The development of novel OMVs that cooperate with other materials or therapeutical agents has a broad range of biomedical applications. Antibiotics, which are usually regarded to be incompatible with bacteria, can be loaded by OMVs through the antibiotic efflux mechanism.^[^ ^79^ ^]^ OMVs coated with gold nanoparticles showed significantly improved stability, and a faster and more durable immune response, compared to treatment with OMVs alone. OMVs hybridized with cancer cell membranes exhibit both cancer‐targeting and immunotherapeutic abilities. Coating hollow polydopamine nanoparticles by OMVs/cell hybrid membrane enhanced cancer killing efficacy, taking advantage of the synergistic immunotherapy of OMVs and photothermal effects mediated by polydopamine nanoparticles.^[^ ^80^ ^]^

However, some antigens presenting on MVs are unable to induce immune responses, such as outer surface protein A.^[^ ^81^ ^]^ Meanwhile, the immune response induced by antigens inside MVs lumen may be weaker than that by surface‐exposed antigens.^[^ ^82^ ^]^ Additionally, because of the distinctive compositions in MVs, all MVs can't be compatible with human bodies. For instance, lipopolysaccharide highly expressed in OMVs from Gram‐negative bacteria has the potential to induce over‐inflammation, fever, and even lead to septic shock.^[^ ^83^ ^]^ Thus, detoxification of MVs before use is important to ensure safe and effective applications. One elegant approach to detoxification is to engineer bacteria for producing lower levels of endotoxins. Lipid A is the endotoxic portion of LPS that triggers the host immune response; however, it can also result in lethal endotoxic shock. Regulating lipid A is an effective way to modulate endotoxin levels in MVs. Knocking out genes including *lpxM* and *lpxL1* for modified lipid A region has proven to reduce TLR4 stimulation, attenuate the toxicity of LPS, and minimize endotoxins on MVs.^[^ ^84^ ^]^ Another modality to reduce MVs’ endotoxic activity is incorporating genes including *pagL* and *pagP* to express lipid A modification enzymes.^[^ ^85^ ^]^ Nevertheless, there remains an outstanding need to develop innovative methods for more efficient detoxification of bacterial MVs.

## 3 Emerging Application

### 3.1 Advanced Diagnosis

#### 3.1.1 Biosensor

A microbial biosensor is a novel device used to detect and quantify biological molecules by converting the biological response into a reported output. In recent years, microbial biosensors have become increasingly important due to their advantages, such as being fast, cost‐effective, and accurate in sensing. They can be easily implemented in difficult‐to‐access locations due to their motility and can be self‐renewed and scaled up in industry applications.^[^ ^86^ ^]^ Microbial biosensors are especially promising in the field of medical diagnostics, exhibiting high sensitivity, stability, and reliability.^[^ ^87^, ^88^ ^]^ For example, the organic‐inorganic hybrid nanocomposites (MB@MI) made from *E. coli*‐specific magainin I were developed for ultrasensitive detection of pathogenic bacteria with a wide linear range and a low detection limit.^[^ ^89^ ^]^ The 3D structure of nanocomposites provides large surface areas, offering more active sites to ensure nanomaterials loading and greatly improving sensitivity for detection. Specifically, bacteria were explicitly bound to the aptamers on the electrode surface and MB@MI nanocomposites, and the number of pathogenic bacteria can be detected according to the changes in signals generated on the electrode surface. Additionally, the sensitivity and response speed can be further improved by using magnetotactic bacteria, the living microbes with superparamagnetic nanoparticles inside, for precision medicine.^[^ ^90^ ^]^

The use of nanomaterials to modify or functionalize electrodes and combine them with microbial biosensors has received a lot of attention. Even though magnetic separation is widely used to separate and detect bacteria from complex matrices with improved sensitivity, the capture efficiency is low which limits its application.^[^ ^91^ ^]^ One reason for this main drawback of microbial biosensors is the delayed electron transport between the microbial cell wall and the electrode surface.^[^ ^92^ ^]^ Fortunately, applying nanomaterials shows promise in optimizing the electron transfer rate, which could expand the application of microbial biosensors.^[^ ^93^, ^94^ ^]^ For example, an electrochemical microbial biosensor was generated by combining metallic nanoparticles with a silk‐derived carbon fiber mat. Carbon fibers immobilized on nanoparticles enabled effective electron tunneling, which enhanced electrical communication between the microbial biosensor and the electrode surface, resulting in higher sensitivity for detecting *E. coli* activities.^[^ ^95^ ^]^ In conclusion, the combination of nanotechnologies and bacteria is believed to provide a unique and robust detecting platform as a novel microbial biosensor, enabling precise diagnosis.

#### 3.1.2 Imaging

**Figure 5:**
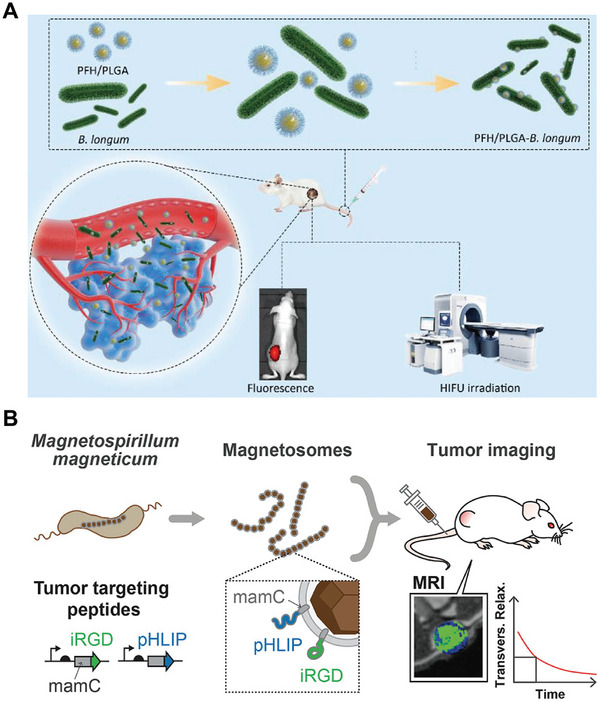
A) Perfluorohexane‐loaded PLGA nanoparticles conjugated with the bacteria of *Bifidobacterium longum* for precision imaging and tumor therapy. Reproduced with permission.^[^ ^98^ ^]^ Copyright 2019, Elsevier B.V. B) Functionalized magnetic nanoparticles from genetically engineered magnetotactic bacteria to enhance MRI contrast for tumor imaging. pHLIP could target the acidic environment of tumor tissues to enhance MRI contrast, while iRGD, an integrin‐binding peptide, could increase binding efficacy. Reproduced with permission.^[^ ^105^ ^]^ Copyright 2020, American Chemical Society.

Imaging technology, such as computed tomography (CT), single‐photon emission computed tomography (PET‐CT), ultrasound, and magnetic resonance, play a critical role in diseases diagnosis and early detection. Nanoparticles are being explored to improve imaging efficacy by enhancing tissue penetration, imaging contrast, and sensitivity. Commonly used nanomaterials include metal chalcogenide quantum dots, single‐walled carbon nanotubes, cerasomal perfluorocarbon nanodroplets, wide‐bandgap semiconducting quantum dots, as well as probes.^[^ ^96^ ^]^ However, these imaging agents often have poor targeting properties and low biocompatibility.^[^ ^97^ ^]^ To solve this problem, the convergence of bacteria and imaging agents enables accumulation in specific tissues, providing higher resolution and sensitivity for diagnosis and therapeutic guidance. Perfluorohexane liquid has a low boiling point and can affect the acoustic environment during high intensity, focused ultrasound irradiation after the liquid‐gas phase transition. Anaerobic bacteria with propensity for colonizing in hypoxic environments could deliver PLGA nanoparticles that encapsulate perfluorohexane liquid to tumor regions, showing a better targeting effect and longer retention time compared to bare nanoparticles (**Figure** **5**A).^[^ ^98^ ^]^ As for the challenges of lacking biodegradability, gas vesicles, a type of air‐filled protein nanostructures initially generated by bacteria, have been developed as degradable nanoscale all‐protein contrast agents. Once internalized by macrophages, gas vesicles will be degraded in the lysosome, resulting in the loss of ultrasonic contrast for visualizing and measuring phagocytic and lysosomal activities.^[^ ^99^ ^]^

Iron‐oxide nanoparticles are a well‐studied contrast agent for magnetic resonance imaging (MRI), a powerful tool for diagnostic imaging. Several coating methods, that use biomaterials such as PEG and human serum albumin, have been developed to improve their biocompatibility and dispersibility in biological fluids.^[^ ^100^ ^]^ Different from traditional coating methods, magnetosomes derived from magnetotactic bacteria show great potential as contrast‐enhancing agents for iron‐oxide nanoparticles in MRI, due to their superparamagnetic properties and extremely high T2 relaxation times, which are necessary for supersensitive MRI.^[^ ^101^ ^]^ This type of iron‐oxide core has a narrow size distribution and is larger than typical iron‐oxide nanoparticles, resulting in high contrast.^[^ ^102^ ^]^ Meanwhile, the size and shape of magnetosomes are strain‐dependent and can be artificially modulated. The lipid bilayers of the magnetosome membrane also provide excellent biocompatibility and multiple functional groups that can be modified for enhanced imaging and therapy.^[^ ^103^ ^]^ For instance, RGD peptide (Arg‐Gly‐Asp) that targets and binds to α_ν_β_3_ integrin on tumor cells can be fused on the surfaces of magnetosomes. This allows the RGD‐labeled magnetosomes to accumulate in tumor tissues and specifically enhance tumor contrast for MRI.^[^ ^104^ ^]^ Multiple peptides of different functions can be genetically engineered for magnetosomes simultaneously, depending on the necessity for imaging in complex contexts. In another study, RGD is designed to bind to the α_ν_β_3_ integrin, while pH low insertion peptide (pHLIP) targets the extracellular acidity associated with tumors, exhibiting enhanced tumor imaging efficiency (Figure 5B).^[^ ^105^ ^]^ Besides MRI, optical imaging is also applied for disease detection. Interestingly, the fluorescence of Rhodamine B is suppressed when conjugating with magnetosomes due to their magnetic property. However, fluorescence is restored after dissociation under external energy or chemical stimuli.^[^ ^106^ ^]^ Thus, a more sensitive fluorescent nanoprobe can be formed by combining magnetosomes with a fluorescent moiety, allowing for monitoring of biological processes or drug release, as well as for tumor imaging in the presence of an external magnetic field. In addition to developing methods with improved sensitivity and contrast, evaluating the biocompatibility and cytotoxicity of magnetosomes is crucial for their application in vivo imaging. Concerns persist regarding the cytokine release and pyrogenicity induced by the endotoxins. While some studies have indicated high blood compatibility and assessed the fate of magnetosomes in interactions with mammalian cells, a thorough and comprehensive safety assessment is indispensable for ensuring the suitability of magnetosomes for in vivo diagnosis.^[^ ^107^ ^]^

### 3.2 Advanced Therapy

#### 3.2.1 Cancer Therapy

##### Immunotherapy

**Figure 6:**
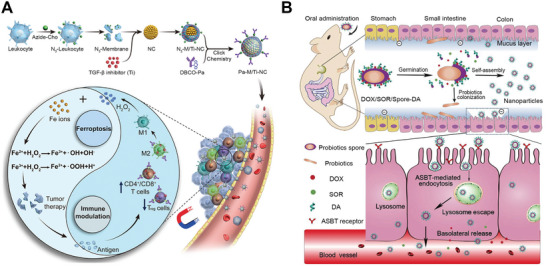
Convergence of bacteria‐derived vesicles with nanoparticles for antitumor therapy. A) TGF‐β inhibitor and PD‐1 antibody‐loaded magnetosome for ferroptosis/immunomodulation synergism in cancer treatment. Reproduced with permission.^[^ ^112^ ^]^ Copyright 2019, American Chemical Society. B) Schematic illustration of deoxycholic acid modified and chemotherapeutics loaded spores for cancer therapy by modulating microbiota and promoting chemotherapeutics absorption via autonomously generated nanoparticles. Reproduced with permission.^[^ ^120^ ^]^ Copyright 2019, Wiley‐VCH.

Immunotherapy is an emerging cancer therapy that harnesses patients’ immune systems for disease treatments. Bacteria combined with nanotechnology also demonstrates tremendous potential for immunotherapy. For example, immune activation of macrophages and leukocytes can be safely induced by attenuated strains of bacteria that work as foreign invaders. Simultaneously, drug‐loaded nanoparticles can compensate for their performing blood circulation and further drive more durable and precise immune response.^[^ ^108^ ^]^ For instance, drug‐loaded polymeric micelles coated with OMVs work as nanomedicine for immunotherapy. OMVs could also be coated with shells from various materials such as polyethylene glycol (PEG) or calcium phosphate. The protective shells could be designed to disintegrate under specific conditions within the tumor, reducing damage to healthy tissues. This disintegration also allows for the administration of higher dosages for better antitumor effects.^[^ ^109^ ^]^ OMVs biofunctionalized with tumor‐targeting peptides have tumor‐targeting capabilities, while the anti‐tumor drug inside, tegafur, directly destroys tumors and sensitizes tumor cells, resulting in a stronger immunostimulatory effect.^[^ ^110^ ^]^ Artificially creating the immunogenic microenvironment for immunomodulation is also a robust strategy, which is accessible by loading various molecules, such as transforming growth factor beta (TGF‐β) inhibitors and programmed cell death protein 1 (PD‐1) antibodies that promote the recruitment of T effector cells, and anti‐tumor M1 macrophages in the bacteria‐based immunotherapy. Combined with photothermal therapy, bacterial OMVs are also harnessed for synergistic systemic immunotherapy. They induce immunogenic cell death when exposed to near‐infrared light and serve as immune adjuvants by promoting dendritic cell maturation.^[^ ^111^ ^]^ Importantly, immunotherapy can also synergize with other modulation of important biological processes, inducing ferroptosis, for example, induces stronger anti‐tumor therapeutic benefits in numerous tumor models (**Figure** **6**A).^[^ ^112^ ^]^

The ability to activate innate or adaptive immunity indicates that bacteria or bacteria‐derived vesicles could also be utilized for vaccines. For example, Hu et al. formed PEI/DNA nanoparticles that can self‐assemble on the surface of bacteria to create a bacteria‐nanoparticle hybrid for DNA vaccine delivery.^[^ ^20^ ^]^ The bacteria could carry nanoparticles and be identified and internalized by macrophages. The loaded plasmids on the bacterial surface could easily escape from the phagosome for antigen expression. The hybrid substantially stimulated T cells and induced cytokine production to inhibit tumor angiogenesis and necrosis. Moreover, to enhance adaptive immune response while reducing undesirable side effects, inactivated bacteria can be replaced by using a bacterial membrane that maintains immunogenicity with adjuvants. In one case, hybrid membranes of tumor and bacteria and encapsulating PLGA nanoparticles can simultaneously deliver tumor antigens and adjuvants into DCs to activate innate and adaptive immunity. Components in the bacterial membrane acted as danger signals, stimulating natural immune responses by interacting with pattern recognition receptors on immune cells, while the tumor cell membrane containing a high concentration of antigenic motifs could trigger systemic immune response. The PLGA core makes it possible to form the highly stabilized hybrid membrane structure, enabling significant tumor‐specific immune responses for long‐term protection against tumor recurrence.^[^ ^13^ ^]^

##### Cancer‐Targeting Therapy

Even though PEGylated or cell membrane‐coated nanoparticles have relatively long retention and provide efficient tumor targeting due to the enhanced permeability and retention effect (EPR) of nanoparticles, the proportion of nanoparticles deposited in tumors remains low, particularly in hypoxic tumor regions. This is because nanoparticles rely on systemic circulation which is a passive process for penetrating tumor tissues. Bacteria with tropism toward various physiological signals/stimuli provide nanoparticles a ride under external control to migrate toward particular locations inside the body and are an important carrier in drug delivery systems.^[^ ^113^ ^]^ The targeting ability of bacteria to the tumor sites relies on several mechanisms, including attaching to tumor vascular, chemotaxis, recruitment by pro‐inflammatory signals, and hypoxia tropism.^[^ ^114^ ^]^ For example, an intracellular bacterium—attenuated *Salmonella typhimurium* (VNP) can be developed as a delivery system to initiate the tumor cell pyroptosis. This design takes advantage of the tumor‐selectivity, mobility, and intracellular targeting ability of bacteria.^[^ ^115^ ^]^ Among all the taxis, magnetotaxis is currently preferred because the location of magnetotactic bacteria targeting tumor tissues can be accurately determined and controlled by an external magnetic source.^[^ ^116^ ^]^ In addition to magnetotactic bacteria, anaerobic bacteria can also combine with functional nanoparticles for hypoxia‐related cancer therapy. Delivering Au NR‐anti‐*C. difficile* by clostridia species of *Clostridium difficile* to hypoxic tumor tissues resulting in converting spatially specific near‐infrared light for heat‐killing effect.^[^ ^117^ ^]^ However, the harsh environment in the gastrointestinal tract might restrict the applications of designing bacterial‐based cancer targeting therapy. Luckily, spores, another dormant life form of bacteria, are encased in a thick hydrophobic protein shell that can withstand the harsh acidic environment, toxins, and high temperatures.^[^ ^118^ ^]^ Notably, after disintegrating the hydrophobic protein shell in the intestines, the spores can germinate into probiotics for regulating intestinal flora balance, thereby making them excellent drug carriers instead of bacteria for oral drug delivery.^[^ ^119^ ^]^ Song et al. constructed an oral autonomous nanoparticles generator by modifying spores with deoxycholic acid and chemotherapeutic medicines doxorubicin and sorafenib for colon cancer therapy.^[^ ^120^ ^]^ The disassociated hydrophobic proteins and hydrophilic drugs could be self‐assembled into nanoparticles with deoxycholic acid decorated on the surface. This increased the internalization of the nanoparticles via apical‐sodium‐dependent bile acid transporter‐mediated endocytosis to enhance the efficiency of intestinal epithelial absorption and improve anti‐tumor therapeutic effect (Figure 6B). Collectively, different from strategies that target specific proteins or surface antigens overexpressed on tumor cells, the bacteria taxis‐mediated targeting offers a wider range of precise possibilities for cancer therapy.

#### 3.2.2 Infectious Diseases

**Figure 7:**
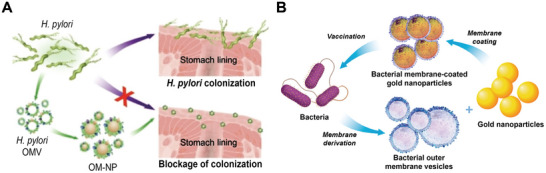
Convergence of bacteria and nanoparticles against infectious diseases. A) *H. pylori* outer membrane encapsulated nanoparticles (OM‐NPs) competitively reduce pathogenic *H. pylori* adhesion against *H. pylori* infections. Reproduced with permission.^[^ ^124^ ^]^ Copyright 2019, Wiley‐VCH. B) Bacterial membrane‐coated gold nanoparticles act as an antibacterial vaccine against the source bacteria. Reproduced with permission.^[^ ^80^ ^]^ Copyright 2015, American Chemical Society.

The first step in bacterial infections is the adhesion of pathogens to host cells or tissues. Adhering to the host helps pathogens resist the body's cleaning processes, allowing them to reach a density level where an infection can start. Therefore, anti‐adhesion therapy is an effective way to prevent and treat infectious diseases and may help to reduce resistance.^[^ ^121^ ^]^ Bacterial adhesins, which identify mucosal receptors, are responsible for pathogen adherence. Many molecules, such as oligosaccharides, have recently been discovered to target adhesins and inhibit pathogen attachment.^[^ ^122^ ^]^ However, pathogen adherence is a complex process involving numerous molecular interactions, making it difficult to eliminate infections thoroughly with single‐target suppression. Bacterial membrane‐encapsulated nanoparticles, with natural bacterial adhesins on their surfaces, can act as decoys to compete with pathogenic bacteria for binding sites. This prevents pathogens from attaching to the host, a process known as competitive exclusion. Moreover, drugs, such as antibiotics, can be loaded into nanoparticles to improve therapeutic efficacy. *Helicobacter pylori* (H. pylori), for example, is well‐adapted to colonize the human stomach, and infections with *H. pylori* can cause serious gastric disorders ranging from chronic gastritis and ulceration to gastric adenocarcinoma.^[^ ^123^ ^]^ *H. pylori* outer membrane encapsulated nanoparticles (OM‐NPs) with similar adhesins on the surface to those of the intact bacteria are capable of interacting with gastric epithelial cells and competitively reducing pathogenic *H. pylori* adhesion. *H. pylori* pathogens pre‐bound to epithelial monolayers could be detached by OM‐NPs to protect against *H. pylori* infections (**Figure** **7**A).^[^ ^124^ ^]^ Alternatively, gastric epithelial cell membrane encapsulated antibiotic‐loaded nanoparticles exhibit intrinsic adhesion to *H. pylori* bacteria and could reduce pathogen colonization on epithelial mucosa by attaching to *H. pylori* pathogens. Meanwhile, the antibiotic carried by nanoparticles could kill pathogens directly due to the *H. pylori* targeting effect.^[^ ^125^ ^]^ Overall, as host‐pathogen adhesion is a common biological event for many pathogenic bacteria, anti‐adhesion therapy based on nanotherapeutic strategies utilizing both bacteria and host cell membranes is promising for infectious disease treatment.

Besides adhesins, bacterial membranes contain many immunogenic antigens with intrinsic adjuvant properties. Thus, bacterial membrane encapsulated nanoparticles that imitate a pathogen could serve as an antibacterial vaccine and stimulate pathogen‐specific immune response but would not result in infectious diseases. Meanwhile, the nanoparticle core offers adjustable physicochemical characteristics, such as particle size and shape, for efficient antigen presentation to immune cells. For example, OMVs secreted by *E. coli* bacteria were utilized to encapsulate gold nanoparticles to form bacterial membrane‐coated gold nanoparticles (BM‐AuNPs). The BM‐AuNPs quickly activated DCs in nearby draining lymph nodes, prompting bacterial‐specific B‐cell and T‐cell responses. They might also be able to stimulate the synthesis of interferon gamma (INFγ) and Interleukin‐17 (IL‐17) in response to *E. coli* infections (Figure 7B).^[^ ^80^ ^]^ In another scenario, while implant‐related infections take place, a hybrid membrane generated from bacterial OMVs and red blood cells could further mitigate the excessive inflammatory response induced by original OMVs while reversing the immunosuppressive microenvironment around implant‐related infections.^[^ ^126^ ^]^ Consequently, bacterial membrane‐coated nanoparticles appear to be a viable strategy for providing an antibacterial vaccine to effectively treat bacterial infections.

#### 3.2.3 Other Diseases

Due to numerous benefits provided by both bacteria and nanoparticles, the convergence of bacteria and nanoparticles has been applied to treat many other diseases. For example, iron deficiency anemia is a global nutritional disorder that affects human health. When iron oxide nanoparticles were incorporated onto the probiotic bacterium *Lactobacillus fermentum*, the anemia treatment efficacy was greater than when the nanoparticles were administrated alone.^[^ ^127^ ^]^ The probiotics helped the maghemite nanoparticles pass through the stomach and accumulate in the intestines and enabled the iron oxide nanoparticles to be internalized into enterocytes for adequate iron levels to treat anemia.^[^ ^128^ ^]^

Type 1 diabetes is an autoimmune disease that destroys insulin producing β‐cells in the pancreas. Glutamic acid decarboxylase (GAD65) has been identified as a key autoantigen in diabetes. Vaccines based on GAD65 proteins or peptides could be used to prevent type 1 diabetes. However, efficient transportation of GAD65 to the gut mucosa's tolerizing milieu is difficult. Taking advantage of their intestinal colonizing ability, bacteria can be genetically engineered to express GAD65 or to deliver nanoparticles comprising antigens to protect functional β‐cells curing diabetes.^[^ ^129^ ^]^

## 4 Modulate Gut Microbiota with Nanotechnology

Gut microbiota is a large community of microorganisms, mostly commensal bacteria that are non‐pathogenic. It colonizes the gastrointestinal mucosal and plays a vital role in maintaining the host's homeostasis and health.^[^ ^130^ ^]^ In addition to providing essential nutrients such as short‐chain fatty acids and amino acids, gut microbiota helps to protect the host against exogenous pathogenic bacteria infection by competitively preventing pathogens’ colonization and invasion.^[^ ^131^ ^]^ The metabolites of gut microbiota are involved in regulating cellular processes, like chemotaxis, cell proliferation, and apoptosis, and in modulating the immune systems by mediating cross‐talk between gut epithelial and immune cells.^[^ ^132^ ^]^

**Figure 8:**
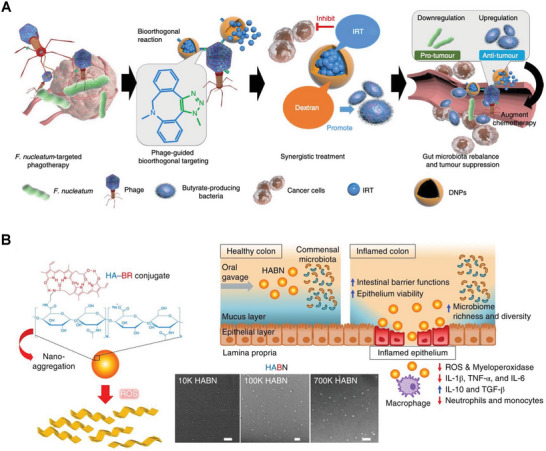
Modulate the gut microbiota with nanoparticles for diverse diseases treatment. A) Schematic illustration of a phage‐guided biotic‐abiotic hybrid nanoplatform for colorectal cancer therapy by modulating gut microbiota and augmenting chemotherapy responses. Reproduced with permission.^[^ ^134^ ^]^ Copyright 2019, Springer Nature. B) Schematic of hyaluronic acid‐bilirubin nanoparticles to regulate gut homeostasis, microbiota, and immune responses for colitis treatment. Reproduced with permission.^[^ ^135^ ^]^ Copyright 2020, Springer Nature.

Dysbiosis of gut microbiota can be caused by exposure to various environmental factors, such as medicines and pathogens, and is also associated with many diseases, like inflammatory bowel disease, diabetes, obesity, and cancers. Oral administration of probiotics and microbiota transplantation are promising approaches to balance gut microbiota to prevent and treat disease. However, the efficacy of these traditional approaches is limited by several barriers, including already crowed bacteria in the intestine which leave little room for transplanted probiotic colonization; the GI tract's harsh environment which impacts the viability and retention of transplanted probiotics; and administering large doses of probiotics into the GI tract might cause adverse side effects. Serving as an alternative to probiotic transplantation, nanoparticles offer an innovative strategy for gut microbiota modulation due to their microscopic and molecular size and biocompatible biomaterial composition.^[^ ^133^ ^]^ Owning to the various roles of gut microbiota in the progression of colorectal cancer, Zheng et al. developed a phage‐guided hybrid nanoplatform combining biotic and abiotic components to modulate gut microbiota for colorectal cancer treatment. In the nanoplatform, phages isolated from saliva could specially target and eliminate the strain of *Fusobacterium nucleatum*, which has been proved to colonize colorectal cancer tissues and promote tumor growth. The dextran nanoparticles had the ability to promote the proliferation of *Clostridium butyricum* strain and stimulate the generation of short‐chain fatty acids. These fatty acids could inhibit tumor growth and induce anti‐tumor immune responses. Additionally, the drug of irinotecan loaded into dextran nanoparticles accumulated in the tumor site, enhancing anti‐tumor efficacy (**Figure** **8**A).^[^ ^134^ ^]^

Inflammatory bowel disease usually involves disorders of the gut microbiota. Traditional therapeutics for inflammatory bowel disease aim to suppress immune responses for alleviating symptoms, which are unable to modulate the balance of gut microbiota or even make it severe. Lee et al. established a nanotherapeutic platform based on hyaluronic acid and bilirubin. Hyaluronic acid can target inflamed colonic epithelium by the interaction between hyaluronic acid and CD44; bilirubin scavenges reactive oxygen species (ROS) to protect colonic epithelial cells from damages by ROS. This nanomedicine not only inhibits overactive immune response but also increases the richness and diversity of gut microbiota and boosts the abundance of strains of *Akkermansia muciniphia* and *Clostridium* XIVα, which are beneficial for the recovery of epithelium barriers (Figure 8B).^[^ ^135^ ^]^ Fullerenol nanoparticles, pectin nanoparticles, and graphene oxide nanoparticles were also reported to modulate the gut microbiota, increase the abundance of short‐chain fatty acids‐producing bacteria, and improve gut morphology for gut microbiota‐associated disease treatment.^[^ ^129^, ^130^, ^131^ ^]^

Prebiotics, such as polysaccharides of inulin, pullulan, and dextran, can stimulate the growth and proliferation of beneficial microorganisms in the gastrointestinal tract, resulting in positive health consequences for the host. Prebiotic nanoparticles could bypass the cellular barriers and be internalized into probiotics for microbiota modulation. For instance, prebiotic nanoparticles prepared by phthalyl inulin or dextran might boost the synthesis of antimicrobial peptide pediocin after being internalized by probiotics of *Pediococcus acidilactidi*. Increased generation of pediocin limits pathogens’ development while increasing beneficial bacteria species that protect against pathogenic diseases.^[^ ^139^, ^140^ ^]^ Overall, prebiotic nanoparticles might be utilized as an alternative to antibiotics in the treatment of infectious diseases.

Nanoparticles are also a great drug delivery system to load drugs and release them in the intestinal tract to modulate gut microbiota. For example, tryptophan‐functionalized chitosan and azobenzene‐functionalized hyaluronic acid encapsulated mesoporous silica nanoparticles could target the gut microbiota due to the existence of azoreductase produced by gut microbiota and tryptophan metabolism ability of microbiota. The payload of cucurbituril on this mesoporous silica nanoparticle could be explicitly released in the intestinal tract and modulate gut homeostasis.^[^ ^141^ ^]^ Nanoparticle curcumin endows curcumin improved solubility and absorbability and can modulate the gut microbiota against colitis.^[^ ^142^ ^]^ However, nanoparticles, such as metal oxide nanoparticles and silver nanoparticles, usually used as food additives would alter gut microbiota populations and impact human health.^[^ ^135^, ^136^, ^137^, ^138^ ^]^ Thus, the principle of safe by design for nanoparticles’ development and application as food ingredients is of great importance.

## 5 Conclusion

Overall, taking advantage of both bacteria and nanoparticles to construct drug delivery systems with unique functionalities provides promises to open new potential frontiers for disease diagnosis and therapy.

First, bacteria can sense, respond, and modulate the gut microenvironment and have distinct properties that make them ideal for being used as intelligent drug delivery vehicles.^[^ ^9^ ^]^ Due to their surface appendages, such as flagella and pili, bacteria can propel themselves in liquid and semi‐solid environments and tend to move forward more favorable conditions to survive. Moreover, bacteria have taxis activities, such as aerotaxis, magnetic taxis, and phototaxis, for responding to a variety of physical and chemical stimuli. Self‐propulsion and taxis properties enable bacteria to be directed to a particular region within the body, which is important in precision medicine. Furthermore, genetically manipulating bacteriaenables the customization of therapeutic action, as well as fine‐tuning and spatiotemporal control, allowing for the construction of unique and sophisticated diagnostic and therapeutic functions that traditional drug delivery systems lack.^[^ ^147^ ^]^ Engineered bacteria also permit in situ protein expression for on‐site synthesis of therapeutic biomolecules, which would substantially eliminate the need for drug purification and significantly reduce the cost of therapy as well as alleviate side effects by concentrating the therapeutics only at disease sites.

The convergence of bacteria with nanoparticles could improve the loading efficiency and diversity of therapeutics and synergize with each other for a broader range of applications. Nanoparticles being used as drug carriers exhibit diverse advantages, including high capacity, high stability, and the ability to incorporate both hydrophilic and hydrophobic molecules. Nanoparticles can also be used to load biomacromolecules, like proteins and peptides, and facilitate controlled or environment‐responsive release, enabling improvement of drug bioavailability. Many strategies have been developed for conjugating nanoparticles on/in bacteria, such as electrostatic interactions, bioconjugation, and covalent binding. The form of nanoparticles, their density, and area of attachment, may change bacterial motility and sensing capabilities, providing adjustable hybrid delivery systems.

Bacteria, however, have many safety concerns that must be addressed prior to their implementation into clinical applications. First, the interaction of bacteria with the host is complicated, and their effects are less predictable than those of small/macromolecular drugs. Thus, bacterial treatment may result in unforeseen consequences, especially when bacteriotherapy is used for different purposes like drug delivery vehicles or immune adjuvants. Precise control over the impact on the immune response following administration is essential. To achieve this, a comprehensive understanding of the functions of components in different bacteria is crucial. Additionally, methods to manipulate these properties serve as a powerful toolbox for tailoring the device in an on‐demand manner. Rigorous examination is also necessary to ensure the appropriate immunostimulatory effect. Secondly, the immune stimulation induced by bacterial lipopolysaccharides or other toxins is still considered dangerous since it can cause septic shock and even mortality. The systemic toxicity induced by PAMPs is also the main obstacle in clinical translation. Moreover, the dosage for bacteria‐based system is difficult to be quantified. If certain strains grow beyond their normal ranges, the balance of microbiota would be disturbed, resulting in dysbiosis and diseases. Given that non‐pathogenic and commensal bacteria are harmless under normal circumstances and interact productively with the host immune system, commensal bacteria may be a more suited choice for bacteria‐based drug delivery systems. However, the dual nature of bacteria in the cancer microenvironment also raises safety concerns, as the commensal bacteria within tumors may have pro‐oncogenic properties. An example is *Fusobacterium nucleatum* (*F. nucleatum*), which has been implicated in the development of colorectal cancer.^[^ ^148^ ^]^ Long‐term evaluation and monitoring of the role of bacteria in tumorigenesis will provide additional insights into determining the stain and dosage for cancer therapy. Effective mitigation strategies are also essential to overcome these safety issues. From a translational standpoint, the formulations incorporating nanoparticles and bacteria have been explored for concrete applications in the pre‐clinical and clinical studies. While the scalability of such platforms benefits from rapid reproduction and mature preparation, the stability of formulations over time and the reproductivity between different batches requires further examination.

Perspectives for future research include designing micro/nanorobots that can access the whole body and provide targeted in vivo diagnostics and therapy with better precision and efficiency. This is also largely driven by the needs of the biomedical community. However, there remain significant technological hurdles in the development of micro/nanorobotic systems within the body, such as biocompatibility, precise motion control, and active force power. The micro/nanorobots based on bacteria and nanoparticles would have multiple unique capabilities, such as controlled navigation, regulated cargo loading and release, and powerful tissue penetration, which are unmet challenges of current drug delivery systems. The precise regulation could be potentially harnessed and boosted by integrating the technologies in the fields of artificial intelligence and synthetic biology, especially for more complex systems.^[^ ^149^ ^]^ Thus, bacteria‐nanoparticle hybrids represent a novel delivery vehicle that can overcome the challenges of traditional delivery platforms and precisely transport payloads to targeted locations, minimizing systemic side effects, boosting therapeutic efficacy, and holding promise for a wide range of biomedical applications.

## Conflict of Interest

The authors declare no conflict of interest.

## Author Contributions

J.L. and S.Y. contributed equally to this work. J. Liu: conceptualized the idea for the study and wrote the original draft. S.Y. wrote the original draft, and reviewed and edited the final manuscript. A.B. reviewed and edited the final manuscript. Q.H. conceptualized the idea for the study; performed supervision; performed funding acquisition, and reviewed and edited the final manuscript.

## References

[1] M. E. Griffin , J. Espinosa , J. L. Becker , J.‐D. Luo , T. S. Carroll , J. K. Jha , G. R. Fanger , H. C. Hang , Science 2021, 373, 1040. 34446607 10.1126/science.abc9113 PMC9503018

[2] M. Luu , Z. Riester , A. Baldrich , N. Reichardt , S. Yuille , A. Busetti , M. Klein , A. Wempe , H. Leister , H. Raifer , F. Picard , K. Muhammad , K. Ohl , R. Romero , F. Fischer , C. A. Bauer , M. Huber , T. M. Gress , M. Lauth , S. Danhof , T. Bopp , T. Nerreter , I. E. Mulder , U. Steinhoff , M. Hudecek , A. Visekruna , Nat. Commun. 2021, 12, 4077. 34210970 10.1038/s41467-021-24331-1 PMC8249424

[3] Y. Xiao , M. T. Angulo , S. Lao , S. T. Weiss , Y.‐Y. Liu , Nat. Commun. 2020, 11, 3329. 32620839 10.1038/s41467-020-17180-x PMC7334230

[4] K. Korpela , O. Helve , K.‐L. Kolho , T. Saisto , K. Skogberg , E. Dikareva , V. Stefanovic , A. Salonen , S. Andersson , W. M. de Vos , Cell 2020, 183, 324. 33007265 10.1016/j.cell.2020.08.047

[5] S. Gude , E. Pinçe , K. M. Taute , A.‐B. Seinen , T. S. Shimizu , S. J. Tans , Nature 2020, 578, 588. 32076271 10.1038/s41586-020-2033-2

[6] G. Vizsnyiczai , G. Frangipane , S. Bianchi , F. Saglimbeni , D. Dell'Arciprete , R. Di Leonardo , Nat. Commun. 2020, 11, 2340. 32393772 10.1038/s41467-020-15711-0 PMC7214458

[7] J. Yang , R. Chawla , K. Y. Rhee , R. Gupta , M. D. Manson , A. Jayaraman , P. P. Lele , Proc. Natl. Acad. Sci 2020, 117, 6114. 32123098 10.1073/pnas.1916974117 PMC7084101

[8] G.‐F. Luo , W.‐H. Chen , X. Zeng , X.‐Z. Zhang , Chem. Soc. Rev. 2021, 50, 945. 33226037 10.1039/d0cs00152j

[9] Z. Li , Y. Wang , J. Liu , P. Rawding , J. Bu , S. Hong , Q. Hu , Adv. Mater. 2021, 33, 2102580. 10.1002/adma.202102580 34347325

[10] H. Strahl , J. Errington , Annu. Rev. Microbiol. 2017, 71, 519. 28697671 10.1146/annurev-micro-102215-095630

[11] R. B. Patel , M. Ye , P. M. Carlson , A. Jaquish , L. Zangl , B. Ma , Y. Wang , I. Arthur , R. Xie , R. J. Brown , X. Wang , R. Sriramaneni , K. Kim , S. Gong , Z. S. Morris , Adv. Mater. 2019, 31, 1902626. 10.1002/adma.201902626 PMC6810793 31523868

[12] J. Shao , M. Xuan , H. Zhang , X. Lin , Z. Wu , Q. He , Angew. Chem. Int. Ed. 2017, 56, 12935. 10.1002/anie.201706570 28816386

[13] L. Chen , H. Qin , R. Zhao , X. Zhao , L. Lin , Y. Chen , Y. Lin , Y. Li , Y. Qin , Y. Li , S. Liu , K. Cheng , H. Chen , J. Shi , G. J. Anderson , Y. Wu , Y. Zhao , G. Nie , Sci. Transl. Med. 2021, 13, eabc2816. 34233949 10.1126/scitranslmed.abc2816

[14] C. M. Dundas , D. Demonte , S. Park , Appl. Microbiol. Biotechnol. 2013, 97, 9343. 24057405 10.1007/s00253-013-5232-z

[15] D. Akin , J. Sturgis , K. Ragheb , D. Sherman , K. Burkholder , J. P. Robinson , A. K. Bhunia , S. Mohammed , R. Bashir , Nat. Nanotechnol. 2007, 2, 441. 18654330 10.1038/nnano.2007.149

[16] G. Dietrich , Nat. Nanotechnol. 2007, 2, 394. 18654320 10.1038/nnano.2007.161

[17] M. Howarth , D. J.‐F. Chinnapen , K. Gerrow , P. C. Dorrestein , M. R. Grandy , N. L. Kelleher , A. El‐Husseini , A. Y. Ting , Nat. Methods 2006, 3, 267. 16554831 10.1038/NMETHXXX PMC2576293

[18] J. M. Lee , J. A. Kim , T.‐C. Yen , I. H. Lee , B. Ahn , Y. Lee , C.‐L. Hsieh , H. M. Kim , Y. Jung , Angew. Chem. Int. Ed Engl. 2016, 55, 3393. 26833545 10.1002/anie.201510885

[19] M. Kojima , Z. Zhang , M. Nakajima , T. Fukuda , Biomed. Microdevices 2012, 14, 1027. 23053448 10.1007/s10544-012-9711-2

[20] Q. Hu , M. Wu , C. Fang , C. Cheng , M. Zhao , W. Fang , P. K. Chu , Y. Ping , G. Tang , Nano Lett 2015, 15, 2732. 25806599 10.1021/acs.nanolett.5b00570

[21] M. Martín , V. Garcés , J. M. Domínguez‐Vera , N. Gálvez , RSC Adv. 2016, 6, 95220.

[22] T. Vangijzegem , D. Stanicki , S. Laurent , Expert Opin. Drug Deliv. 2019, 16, 69. 30496697 10.1080/17425247.2019.1554647

[23] M. Kahraman , A. I. Zamaleeva , R. F. Fakhrullin , M. Culha , Anal. Bioanal. Chem. 2009, 395, 2559. 19795108 10.1007/s00216-009-3159-0

[24] A. C. Anselmo , K. J. McHugh , J. Webster , R. Langer , A. Jaklenec , Adv. Mater. 2016, 28, 9486. 27616140 10.1002/adma.201603270 PMC5287492

[25] J. Liu , Y. Wang , W. J. Heelan , Y. Chen , Z. Li , Q. Hu , Sci. Adv. 2022, 8, eabp8798. 36367930 10.1126/sciadv.abp8798 PMC9651739

[26] H. Zhang , Y. Wang , L. Zhu , Z. Qi , K. Cao , J. Chang , L. Hou , J. Controlled Release 2023, 360, 660. 10.1016/j.jconrel.2023.07.014 37433371

[27] S. Taherkhani , M. Mohammadi , J. Daoud , S. Martel , M. Tabrizian , ACS Nano 2014, 8, 5049. 24684397 10.1021/nn5011304

[28] O. Felfoul , M. Mohammadi , S. Taherkhani , D. de Lanauze , Y. Z Xu , D. Loghin , S. Essa , S. Jancik , D. Houle , M. Lafleur , L. Gaboury , M. Tabrizian , N. Kaou , M. Atkin , T. Vuong , G. Batist , N. Beauchemin , D. Radzioch , S. Martel , Nat. Nanotechnol. 2016, 11, 941. 27525475 10.1038/nnano.2016.137 PMC6094936

[29] V. M. Moreno , E. Álvarez , I. Izquierdo‐Barba , A. Baeza , J. Serrano‐López , M. Vallet‐Regí , Adv. Mater. Interfaces 2020, 7, 1901942. 33154882 10.1002/admi.201901942 PMC7116290

[30] H. Luo , Y. Chen , X. Kuang , X. Wang , F. Yang , Z. Cao , L. Wang , S. Lin , F. Wu , J. Liu , Nat. Commun. 2022, 13, 7808. 36528693 10.1038/s41467-022-35579-6 PMC9759558

[31] X.‐N. Wang , M.‐T. Niu , J.‐X. Fan , Q.‐W. Chen , X.‐Z. Zhang , Nano Lett 2021, 21, 4270. 33955768 10.1021/acs.nanolett.1c00408

[32] K. K. Sakimoto , A. B. Wong , P. Yang , Science 2016, 351, 74. 26721997 10.1126/science.aad3317

[33] S. Yan , X. Zeng , Y. Wang , B.‐F. Liu , Adv. Healthcare Mater. 2020, 9, e2000046. 10.1002/adhm.202000046 32400080

[34] F. Ghibaudo , E. Gerbino , G. J. Copello , V. Campo Dall’ Orto , A. Gómez‐Zavaglia , Colloids Surf. B Biointerfaces 2019, 180, 193. 31054459 10.1016/j.colsurfb.2019.04.049

[35] E. Firlar , M. Ouy , A. Bogdanowicz , L. Covnot , B. Song , Y. Nadkarni , R. Shahbazian‐Yassar , T. Shokuhfar , Nanoscale 2019, 11, 698. 30565643 10.1039/c8nr08647h

[36] S. Tong , C. A. Quinto , L. Zhang , P. Mohindra , G. Bao , ACS Nano 2017, 11, 6808. 28625045 10.1021/acsnano.7b01762

[37] S. Sturm , M. Siglreitmeier , D. Wolf , K. Vogel , M. Gratz , D. Faivre , A. Lubk , B. Büchner , E. V. Sturm (née Rosseeva) , H. Cölfen , Adv. Funct. Mater. 2019, 29, 1905996.

[38] A. Arakaki , M. Goto , M. Maruyama , T. Yoda , M. Tanaka , A. Yamagishi , Y. Yoshikuni , T. Matsunaga , Biotechnol. J. 2020, 15, 2000278. 10.1002/biot.202000278 32846013

[39] M. Furubayashi , A. K. Wallace , L. M. González , J. P. Jahnke , B. M. Hanrahan , A. L. Payne , D. N. Stratis‐Cullum , M. T. Gray , H. Liu , M. K. Rhoads , C. A. Voigt , Adv. Funct. Mater. 2021, 31, 2004813.

[40] R. Uebe , B. Voigt , T. Schweder , D. Albrecht , E. Katzmann , C. Lang , L. Böttger , B. Matzanke , D. Schüler , J. Bacteriol. 2010, 192, 4192. 20562310 10.1128/JB.00319-10 PMC2916424

[41] F. Zhang , L. Zhao , S. Wang , J. Yang , G. Lu , N. Luo , X. Gao , G. Ma , H.‐Y. Xie , W. Wei , Adv. Funct. Mater. 2018, 28, 1703326.

[42] C.‐X. Li , Y. Zhang , Y.‐D. Qi , M.‐D. Liu , B. Li , M.‐K. Zhang , J. Feng , X.‐Z. Zhang , Adv. Ther. 2021, 4, 2000231.

[43] F. Li , W. Nie , F. Zhang , G. Lu , C. Lv , Y. Lv , W. Bao , L. Zhang , S. Wang , X. Gao , W. Wei , H.‐Y. Xie , ACS Cent. Sci. 2019, 5, 796. 31139716 10.1021/acscentsci.9b00060 PMC6535768

[44] Q. Zhang , W. Wei , P. Wang , L. Zuo , F. Li , J. Xu , X. Xi , X. Gao , G. Ma , H. Xie , ACS Nano 2017, 11, 10724. 28921946 10.1021/acsnano.7b04955

[45] J. A. MacDiarmid , J. Madrid‐Weiss , N. B. Amaro‐Mugridge , L. Phillips , H. Brahmbhatt , Cell Cycle Georget. Tex 2007, 6, 2099. 10.4161/cc.6.17.4648 17786046

[46] D. T. T. Vinh , N. T. H. Khue , J. Appl. Pharm. Sci. 2013, 3, 33.

[47] Y. Zhang , W. Ji , L. He , Y. Chen , X. Ding , Y. Sun , S. Hu , H. Yang , W. Huang , Y. Zhang , F. Liu , L. Xia , Theranostics 2018, 8, 1690. 29556350 10.7150/thno.21575 PMC5858176

[48] J. A. MacDiarmid , N. B. Mugridge , J. C. Weiss , L. Phillips , A. L. Burn , R. P. Paulin , J. E. Haasdyk , K.‐A. Dickson , V. N. Brahmbhatt , S. T. Pattison , A. C. James , G. Al Bakri , R. C. Straw , B. Stillman , R. M. Graham , H. Brahmbhatt , Cancer Cell 2007, 11, 431. 17482133 10.1016/j.ccr.2007.03.012

[49] A. Flemming , Nat. Rev. Drug Discov. 2007, 6, 519.

[50] B. J. Solomon , J. Desai , M. Rosenthal , G. A. McArthur , S. T. Pattison , S. L. Pattison , J. MacDiarmid , H. Brahmbhatt , A. M. Scott , PLoS ONE 2015, 10, e0144559. 26659127 10.1371/journal.pone.0144559 PMC4699457

[51] J. R. Whittle , J. D. Lickliter , H. K. Gan , A. M. Scott , J. Simes , B. J. Solomon , J. A. MacDiarmid , H. Brahmbhatt , M. A. Rosenthal , J. Clin. Neurosci. Off. J. Neurosurg. Soc. Australas. 2015, 22, 1889. 10.1016/j.jocn.2015.06.005 26279503

[52] M. Jivrajani , M. Nivsarkar , Methods Mol. Biol. Clifton NJ 2019, 1974, 111. 10.1007/978-1-4939-9220-1_9 31098999

[53] M. Jivrajani , M. Nivsarkar , Nanomed. Nanotechnol. Biol. Med. 2016, 12, 2485. 10.1016/j.nano.2016.06.004 27378204

[54] P. Gemski , D. E. Griffin , Infect. Immun. 1980, 30, 297. 7002790 10.1128/iai.30.1.297-302.1980 PMC551307

[55] G. R. Barker , C. S. Cordery , D. Jackson , S. F. Le Grice , J. Gen. Microbiol. 1979, 111, 387. 383890 10.1099/00221287-111-2-387

[56] M. Jivrajani , N. Shrivastava , M. Nivsarkar , J. Microbiol. Methods 2013, 92, 340. 23234883 10.1016/j.mimet.2012.12.002

[57] J.‐Y. Lee , H. E. Choy , J.‐H. Lee , G.‐J. Kim , J. Microbiol. Biotechnol. 2015, 25, 554. 25341464 10.4014/jmb.1408.08037

[58] S.‐Y. Park , J.‐Y. Lee , W.‐S. Chang , H. E. Choy , G.‐J. Kim , J. Microbiol. Methods 2011, 86, 108. 21504766 10.1016/j.mimet.2011.04.003

[59] M. Toyofuku , N. Nomura , L. Eberl , Nat. Rev. Microbiol. 2019, 17, 13. 30397270 10.1038/s41579-018-0112-2

[60] D. G. Bishop , E. Work , Biochem. J. 1965, 96, 567. 4953781 10.1042/bj0960567 PMC1207076

[61] K. W. Knox , M. Vesk , E. Work , J. Bacteriol. 1966, 92, 1206. 4959044 10.1128/jb.92.4.1206-1217.1966 PMC276396

[62] E.‐Y. Lee , D.‐Y. Choi , D.‐K. Kim , J.‐W. Kim , J. O. Park , S. Kim , S.‐H. Kim , D. M. Desiderio , Y.‐K. Kim , K.‐P. Kim , Y. S. Gho , Proteomics 2009, 9, 5425. 19834908 10.1002/pmic.200900338

[63] C. Schwechheimer , M. J. Kuehn , Nat. Rev. Microbiol. 2015, 13, 605. 26373371 10.1038/nrmicro3525 PMC5308417

[64] M. Kaparakis‐Liaskos , R. L. Ferrero , Nat. Rev. Immunol. 2015, 15, 375. 25976515 10.1038/nri3837

[65] M. Toyofuku , Y. Tashiro , H. Yusuke , M. Kurosawa , N. Nomura , Adv. Colloid Interface Sci. 2015, 226, 65. 26422802 10.1016/j.cis.2015.08.013

[66] M. Toyofuku , G. Cárcamo‐Oyarce , T. Yamamoto , F. Eisenstein , C.‐C. Hsiao , M. Kurosawa , K. Gademann , M. Pilhofer , N. Nomura , L. Eberl , Nat. Commun. 2017, 8, 481. 28883390 10.1038/s41467-017-00492-w PMC5589764

[67] Y. Shen , M. L. Giardino Torchia , G. W. Lawson , C. L. Karp , J. D. Ashwell , S. K. Mazmanian , Cell Host Microbe 2012, 12, 509. 22999859 10.1016/j.chom.2012.08.004 PMC3895402

[68] R. Stentz , S. Osborne , N. Horn , A. W. H. Li , I. Hautefort , R. Bongaerts , M. Rouyer , P. Bailey , S. B. Shears , A. M. Hemmings , C. A. Brearley , S. R. Carding , Cell Rep 2014, 6, 646. 24529702 10.1016/j.celrep.2014.01.021 PMC3969271

[69] S. K. Vanaja , A. J. Russo , B. Behl , I. Banerjee , M. Yankova , S. D. Deshmukh , V. A. K. Rathinam , Cell 2016, 165, 1106. 27156449 10.1016/j.cell.2016.04.015 PMC4874922

[70] T. N. Ellis , S. A. Leiman , M. J. Kuehn , Infect. Immun. 2010, 78, 3822. 20605984 10.1128/IAI.00433-10 PMC2937433

[71] M. J. H. Gerritzen , D. E. Martens , R. H. Wijffels , L. van der Pol , M. Stork , Biotechnol. Adv. 2017, 35, 565. 28522212 10.1016/j.biotechadv.2017.05.003

[72] K. Cheng , R. Zhao , Y. Li , Y. Qi , Y. Wang , Y. Zhang , H. Qin , Y. Qin , L. Chen , C. Li , J. Liang , Y. Li , J. Xu , X. Han , G. J. Anderson , J. Shi , L. Ren , X. Zhao , G. Nie , Nat. Commun. 2021, 12, 2041. 33824314 10.1038/s41467-021-22308-8 PMC8024398

[73] M. H. Daleke‐Schermerhorn , T. Felix , Z. Soprova , C. M. Ten Hagen‐Jongman , D. Vikström , L. Majlessi , J. Beskers , F. Follmann , K. de Punder , N. N. van der Wel , T. Baumgarten , T. V. Pham , S. R. Piersma , C. R. Jiménez , P. van Ulsen , J.‐W. de Gier , C. Leclerc , W. S. P. Jong , J. Luirink , Appl. Environ. Microbiol. 2014, 80, 5854. 25038093 10.1128/AEM.01941-14 PMC4178611

[74] K. Kuipers , M. H. Daleke‐Schermerhorn , W. S. P. Jong , C. M. ten Hagen‐Jongman , F. van Opzeeland , E. Simonetti , J. Luirink , M. I. de Jonge , Vaccine 2015, 33, 2022. 25776921 10.1016/j.vaccine.2015.03.010

[75] L. Fantappiè , M. de Santis , E. Chiarot , F. Carboni , G. Bensi , O. Jousson , I. Margarit , G. Grandi , J. Extracell. Vesicles 2014, 3, 24015. 10.3402/jev.v3.24015 PMC4131003 25147647

[76] M. Muralinath , M. J. Kuehn , K. L. Roland , R. Curtiss , Infect. Immun. 2011, 79, 887. 21115718 10.1128/IAI.00950-10 PMC3028854

[77] K. Kuerban , X. Gao , H. Zhang , J. Liu , M. Dong , L. Wu , R. Ye , M. Feng , L. Ye , Acta Pharm. Sin. B 2020, 10, 1534. 32963948 10.1016/j.apsb.2020.02.002 PMC7488491

[78] V. Gujrati , S. Kim , S.‐H. Kim , J. J. Min , H. E. Choy , S. C. Kim , S. Jon , ACS Nano 2014, 8, 1525. 24410085 10.1021/nn405724x

[79] W. Huang , Q. Zhang , W. Li , M. Yuan , J. Zhou , L. Hua , Y. Chen , C. Ye , Y. Ma , J. Control. Release Off. J. Control. Release Soc. 2020, 317, 1. 10.1016/j.jconrel.2019.11.017 31738965

[80] W. Gao , R. H. Fang , S. Thamphiwatana , B. T. Luk , J. Li , P. Angsantikul , Q. Zhang , C.‐M. J. Hu , L. Zhang , Nano Lett 2015, 15, 1403. 25615236 10.1021/nl504798g PMC4399974

[81] M. L. M. Salverda , S. M. Meinderts , H.‐J. Hamstra , A. Wagemakers , J. W. R. Hovius , A. van der Ark , M. Stork , P. van der Ley , Vaccine 2016, 34, 1025. 26801064 10.1016/j.vaccine.2016.01.019

[82] S. Barat , Y. Willer , K. Rizos , B. Claudi , A. Mazé , A. K. Schemmer , D. Kirchhoff , A. Schmidt , N. Burton , D. Bumann , PLoS Pathog 2012, 8, e1002966. 23093937 10.1371/journal.ppat.1002966 PMC3475680

[83] Y. M. D. Gnopo , H. C. Watkins , T. C. Stevenson , M. P. DeLisa , D. Putnam , Adv. Drug Deliv. Rev. 2017, 114, 132. 28501509 10.1016/j.addr.2017.05.003

[84] B. D. Needham , S. M. Carroll , D. K. Giles , G. Georgiou , M. Whiteley , M. S. Trent , Proc. Natl. Acad. Sci 2013, 110, 1464. 23297218 10.1073/pnas.1218080110 PMC3557076

[85] B. D. Needham , M. S. Trent , Nat. Rev. Microbiol. 2013, 11, 467. 23748343 10.1038/nrmicro3047 PMC6913092

[86] M. E. Inda , T. K. Lu , Annu. Rev. Microbiol. 2020, 74, 337. 32660390 10.1146/annurev-micro-022620-081059

[87] M. M. Hassan , A. Ranzoni , M. A. Cooper , Biosens. Bioelectron. 2018, 99, 150. 28753457 10.1016/j.bios.2017.07.057

[88] S. Sharifi , S. Z. Vahed , E. Ahmadian , S. M. Dizaj , A. Eftekhari , R. Khalilov , M. Ahmadi , E. Hamidi‐Asl , M. Labib , Biosens. Bioelectron. 2020, 150, 111933. 31818764 10.1016/j.bios.2019.111933

[89] S. Bu , K. Wang , Z. Li , C. Wang , Z. Hao , W. Liu , J. Wan , Analyst 2020, 145, 4328. 32367088 10.1039/d0an00470g

[90] A. Dieudonné , S. Prévéral , D. Pignol , Appl. Environ. Microbiol. 2020, 86, e00803. 32385084 10.1128/AEM.00803-20 PMC7357492

[91] K. Wu , D. Su , J. Liu , R. Saha , J.‐P. Wang , Nanotechnology 2019, 30, 502003. 31491782 10.1088/1361-6528/ab4241

[92] F. Li , C. Lei , Q. Shen , L. Li , M. Wang , M. Guo , Y. Huang , Z. Nie , S. Yao , Nanoscale 2012, 5, 653. 23223666 10.1039/c2nr32156d

[93] J. Chen , S. M. Andler , J. M. Goddard , S. R. Nugen , V. M. Rotello , Chem. Soc. Rev. 2017, 46, 1272. 27942636 10.1039/c6cs00313c PMC5339056

[94] H. Kumar , K. Kuča , S. K. Bhatia , K. Saini , A. Kaushal , R. Verma , T. C. Bhalla , D. Kumar , Sensors 2020, 20, 1966 32244581 10.3390/s20071966 PMC7181077

[95] J. W. Lim , D. Ha , J. Lee , S. K. Lee , T. Kim , Front. Bioeng. Biotechnol. 2015, 3, 61. 26029689 10.3389/fbioe.2015.00061 PMC4426784

[96] Kenry , Y. Duan , B. Liu , Adv. Mater. Deerfield Beach Fla 2018, 30, e1802394. 10.1002/adma.201802394 30182451

[97] S. Zhu , R. Tian , A. L. Antaris , X. Chen , H. Dai , Adv. Mater. Deerfield Beach Fla 2019, 31, e1900321. 10.1002/adma.201900321 PMC6555689 31025403

[98] Y. Luo , D. Xu , X. Gao , J. Xiong , B. Jiang , Y. Zhang , Y. Wang , Y. Tang , C. Chen , H. Qiao , H. Li , J. Zou , Biochem. Biophys. Res. Commun. 2019, 514, 1147. 31103266 10.1016/j.bbrc.2019.05.074

[99] B. Ling , J. Lee , D. Maresca , A. Lee‐Gosselin , D. Malounda , M. B. Swift , M. G. Shapiro , ACS Nano 2020, 14, 12210. 32902951 10.1021/acsnano.0c05912 PMC7685203

[100] E. Erdal , M. Demirbilek , Y. Yeh , Ö. Akbal , L. Ruff , D. Bozkurt , A. Cabuk , Y. Senel , B. Gumuskaya , O. Algın , S. Colak , S. Esener , E. B. Denkbas , Appl. Biochem. Biotechnol. 2018, 185, 91. 29082480 10.1007/s12010-017-2642-x

[101] A. Kraupner , D. Eberbeck , D. Heinke , R. Uebe , D. Schüler , A. Briel , Nanoscale 2017, 9, 5788. 28447690 10.1039/c7nr01530e

[102] Y. Zhang , Q. Ni , C. Xu , B. Wan , Y. Geng , G. Zheng , Z. Yang , J. Tao , Y. Zhao , J. Wen , J. Zhang , S. Wang , Y. Tang , Y. Li , Q. Zhang , L. Liu , Z. Teng , G. Lu , ACS Appl. Mater. Interfaces 2019, 11, 3654. 30495920 10.1021/acsami.8b15838

[103] C. Chen , S. Wang , L. Li , P. Wang , C. Chen , Z. Sun , T. Song , Biomaterials 2016, 104, 352. 27487574 10.1016/j.biomaterials.2016.07.030

[104] M. Boucher , F. Geffroy , S. Prévéral , L. Bellanger , E. Selingue , G. Adryanczyk‐Perrier , M. Péan , C. T. Lefèvre , D. Pignol , N. Ginet , S. Mériaux , Biomaterials 2017, 121, 167. 28088078 10.1016/j.biomaterials.2016.12.013

[105] S. Schuerle , M. Furubayashi , A. P. Soleimany , T. Gwisai , W. Huang , C. Voigt , S. N. Bhatia , ACS Synth. Biol. 2020, 9, 392. 31922737 10.1021/acssynbio.9b00416 PMC7934227

[106] E. Alphandéry , D. A. Haidar , O. Seksek , F. Guyot , I. Chebbi , Nanoscale 2018, 10, 10918. 29850738 10.1039/c8nr02164c

[107] F. Mickoleit , F. Pfister , B. Friedrich , S. Markert , A. Kerpes , C. Janko , S. Lyer , C. Alexiou , D. Schüler , R. Tietze , ACS Appl. Nano Mater. 2024, 7, 1278.

[108] L. M. Wood , Y. Paterson , Front. Cell. Infect. Microbiol. 2014, 4, 51. 24860789 10.3389/fcimb.2014.00051 PMC4026700

[109] X. Chen , P. Li , B. Luo , C. Song , M. Wu , Y. Yao , D. Wang , X. Li , B. Hu , S. He , Y. Zhao , C. Wang , X. Yang , J. Hu , ACS Nano 2024, 18, 1357. 38164903 10.1021/acsnano.3c05714

[110] Q. Chen , H. Bai , W. Wu , G. Huang , Y. Li , M. Wu , G. Tang , Y. Ping , Nano Lett 2020, 20, 11. 31858807 10.1021/acs.nanolett.9b02182

[111] J. Qin , T. Yang , J. Li , G. Zhan , X. Li , Z. Wei , Z. Chen , W. Zheng , H. Chen , X. Yang , L. Gan , Nano Today 2022, 46, 101591.

[112] F. Zhang , F. Li , G.‐H. Lu , W. Nie , L. Zhang , Y. Lv , W. Bao , X. Gao , W. Wei , K. Pu , H.‐Y. Xie , ACS Nano 2019, 13, 5662. 31046234 10.1021/acsnano.9b00892

[113] H. Pan , M. Zheng , A. Ma , L. Liu , L. Cai , Adv. Mater. 2021, 33, 2100241. 10.1002/adma.202100241 34121236

[114] M. T.‐Q. Duong , Y. Qin , S.‐H. You , J.‐J. Min , Exp. Mol. Med. 2019, 51, 1. 10.1038/s12276-019-0297-0 PMC6906302 31827064

[115] Z. Li , F. Mo , Y. Wang , W. Li , Y. Chen , J. Liu , T.‐J. Chen‐Mayfield , Q. Hu , Nat. Commun. 2022, 13, 6321. 36280674 10.1038/s41467-022-34036-8 PMC9592600

[116] D. Gandia , L. Gandarias , I. Rodrigo , J. Robles‐García , R. Das , E. Garaio , J. Á. García , M.‐H. Phan , H. Srikanth , I. Orue , J. Alonso , A. Muela , M. L. Fdez‐Gubieda , Small Weinh. Bergstr. Ger. 2019, 15, e1902626. 10.1002/smll.201902626 31454160

[117] C.‐H. Luo , C.‐T. Huang , C.‐H. Su , C.‐S. Yeh , Nano Lett 2016, 16, 3493. 27148804 10.1021/acs.nanolett.6b00262

[118] M. A. Neag , A. Catinean , D. M. Muntean , M. R. Pop , C. I. Bocsan , E. C. Botan , A. D. Buzoianu , Nutrients 2020, 12, 632. 32120994 10.3390/nu12030632 PMC7146158

[119] L. R. Weerakkody , C. Witharana , Life Sci 2019, 235, 116839. 31499068 10.1016/j.lfs.2019.116839

[120] Q. Song , C. Zheng , J. Jia , H. Zhao , Q. Feng , H. Zhang , L. Wang , Z. Zhang , Y. Zhang , Adv. Mater. 2019, 31, 1903793. 10.1002/adma.201903793 31490587

[121] A. Asadi , S. Razavi , M. Talebi , M. Gholami , Infection 2019, 47, 13. 30276540 10.1007/s15010-018-1222-5

[122] J. Poole , C. J. Day , M. von Itzstein , J. C. Paton , M. P. Jennings , Nat. Rev. Microbiol. 2018, 16, 440. 29674747 10.1038/s41579-018-0007-2

[123] L. Zhang , L. Zhang , H. Deng , H. Li , W. Tang , L. Guan , Y. Qiu , M. J. Donovan , Z. Chen , W. Tan , Nat. Commun. 2021, 12, 2002 33790299 10.1038/s41467-021-22286-x PMC8012368

[124] Y. Zhang , Y. Chen , C. Lo , J. Zhuang , P. Angsantikul , Q. Zhang , X. Wei , Z. Zhou , M. Obonyo , R. H. Fang , W. Gao , L. Zhang , Angew. Chem. Int. Ed. 2019, 58, 11404. 10.1002/anie.201906280 31206942

[125] P. Angsantikul , S. Thamphiwatana , Q. Zhang , K. Spiekermann , J. Zhuang , R. H. Fang , W. Gao , M. Obonyo , L. Zhang , Adv. Ther. 2018, 1, 1800016. 10.1002/adtp.201800016 PMC6176867 30320205

[126] C. Ding , R. He , T. Cheng , J. Wang , X. Liu , G. Guo , Y. Chen , Adv. Funct. Mater. 2023, 33, 2304168.

[127] V. Garcés , A. Rodríguez‐Nogales , A. González , N. Gálvez , M. E. Rodríguez‐Cabezas , M. L. García‐Martin , L. Gutiérrez , D. Rondón , M. Olivares , J. Gálvez , J. M. Dominguez‐Vera , Bioconjug. Chem. 2018, 29, 1785. 29718659 10.1021/acs.bioconjchem.8b00245

[128] M. Martín , A. Rodríguez‐Nogales , V. Garcés , N. Gálvez , L. Gutiérrez , J. Gálvez , D. Rondón , M. Olivares , J. M. Dominguez‐Vera , Nanoscale 2016, 8, 15041. 27477118 10.1039/c6nr04678a

[129] S. Robert , C. Gysemans , T. Takiishi , H. Korf , I. Spagnuolo , G. Sebastiani , K. Van Huynegem , L. Steidler , S. Caluwaerts , P. Demetter , C. H. Wasserfall , M. A. Atkinson , F. Dotta , P. Rottiers , T. L. Van Belle , C. Mathieu , Diabetes 2014, 63, 2876. 24677716 10.2337/db13-1236

[130] E. Thursby , N. Juge , Biochem. J. 2017, 474, 1823. 28512250 10.1042/BCJ20160510 PMC5433529

[131] A. Stacy , V. Andrade‐Oliveira , J. A. McCulloch , B. Hild , J. H. Oh , P. J. Perez‐Chaparro , C. K. Sim , A. I. Lim , V. M. Link , M. Enamorado , G. Trinchieri , J. A. Segre , B. Rehermann , Y. Belkaid , Cell 2021, 184, 615. 33453153 10.1016/j.cell.2020.12.011 PMC8786454

[132] Y. Fan , O. Pedersen , Nat. Rev. Microbiol. 2021, 19, 55. 32887946 10.1038/s41579-020-0433-9

[133] W. Song , A. C. Anselmo , L. Huang , Nat. Nanotechnol. 2019, 14, 1093. 31802032 10.1038/s41565-019-0589-5

[134] D.‐W. Zheng , X. Dong , P. Pan , K.‐W. Chen , J.‐X. Fan , S.‐X. Cheng , X.‐Z. Zhang , Nat. Biomed. Eng. 2019, 3, 717. 31332342 10.1038/s41551-019-0423-2

[135] Y. Lee , K. Sugihara , M. G. Gillilland , S. Jon , N. Kamada , J. J. Moon , Nat. Mater. 2020, 19, 118. 31427744 10.1038/s41563-019-0462-9 PMC6923573

[136] J. Li , R. Lei , X. Li , F. Xiong , Q. Zhang , Y. Zhou , S. Yang , Y. Chang , K. Chen , W. Gu , C. Wu , G. Xing , Part. Fibre Toxicol. 2018, 15, 5. 29343276 10.1186/s12989-018-0241-9 PMC5773151

[137] H. P. S. U. Chandrarathna , T. D. Liyanage , S. L. Edirisinghe , S. H. S. Dananjaya , E. H. T. Thulshan , C. Nikapitiya , C. Oh , D.‐H. Kang , M. De Zoysa , Mar. Drugs 2020, 18, 175. 32245246 10.3390/md18030175 PMC7143556

[138] J. Li , S. Yang , J. Yu , R. Cui , R. Liu , R. Lei , Y. Chang , H. Geng , Y. Qin , W. Gu , S. Xia , K. Chen , J. Kong , G. Chen , C. Wu , G. Xing , RSC Adv. 2018, 8, 31366. 35548257 10.1039/c8ra06058d PMC9085910

[139] L. Hong , C.‐S. Cho , W.‐S. Kim , Y.‐J. Choi , S.‐K. Kang , J. Appl. Microbiol. 2021, 130, 439. 32500649 10.1111/jam.14735

[140] W.‐S. Kim , G. G. Han , L. Hong , S.‐K. Kang , M. Shokouhimehr , Y.‐J. Choi , C.‐S. Cho , Biomaterials 2019, 218, 119360. 31336278 10.1016/j.biomaterials.2019.119360

[141] S. Cheng , H. Shen , S. Zhao , Y. Zhang , H. Xu , L. Wang , B. Di , L. Xu , C. Hu , Nanoscale 2020, 12, 15348. 32648873 10.1039/d0nr03037f

[142] M. Ohno , A. Nishida , Y. Sugitani , K. Nishino , O. Inatomi , M. Sugimoto , M. Kawahara , A. Andoh , PloS One 2017, 12, e0185999. 28985227 10.1371/journal.pone.0185999 PMC5630155

[143] Z. Chen , S. Han , D. Zhou , S. Zhou , G. Jia , Nanoscale 2019, 11, 22398. 31738363 10.1039/c9nr07580a

[144] N. Kolba , Z. Guo , F. M. Olivas , G. J. Mahler , E. Tako , Food Chem. Toxicol. Int. J. Publ. Br. Ind. Biol. Res. Assoc. 2020, 135, 110896. 10.1016/j.fct.2019.110896 PMC8985309 31654707

[145] K. Williams , J. Milner , M. D. Boudreau , K. Gokulan , C. E. Cerniglia , S. Khare , Nanotoxicology 2015, 9, 279. 24877679 10.3109/17435390.2014.921346

[146] S. Siemer , A. Hahlbrock , C. Vallet , D. J. McClements , J. Balszuweit , J. Voskuhl , D. Docter , S. Wessler , S. K. Knauer , D. Westmeier , R. H. Stauber , NPJ Sci. Food 2018, 2, 22. 30882042 10.1038/s41538-018-0030-8 PMC6420113

[147] I. Y. Hwang , E. Koh , A. Wong , J. C. March , W. E. Bentley , Y. S. Lee , M. W. Chang , Nat. Commun. 2017, 8, 15028. 28398304 10.1038/ncomms15028 PMC5394271

[148] X. Zheng , R. Liu , C. Zhou , H. Yu , W. Luo , J. Zhu , J. Liu , Z. Zhang , N. Xie , X. Peng , X. Xu , L. Cheng , Q. Yuan , C. Huang , X. Zhou , Cancer Res. 2021, 81, 6157. 34645607 10.1158/0008-5472.CAN-21-2273 PMC9397643

[149] I. Zanella , E. König , M. Tomasi , A. Gagliardi , L. Frattini , L. Fantappiè , C. Irene , F. Zerbini , E. Caproni , S. J. Isaac , M. Grigolato , R. Corbellari , S. Valensin , I. Ferlenghi , F. Giusti , L. Bini , Y. Ashhab , A. Grandi , G. Grandi , J. Extracell. Vesicles 2021, 10, e12066. 33643549 10.1002/jev2.12066 PMC7886703

